# Supplementary figures and images for: Stabilization of GTSE1 by cyclin D1–CDK4/6-mediated phosphorylation promotes cell proliferation with implications for cancer prognosis
Source: eLife. 2025 Apr 24;13:RP101075. doi: 10.7554/eLife.101075 (PMC12021411; doi:10.7554/eLife.101075)

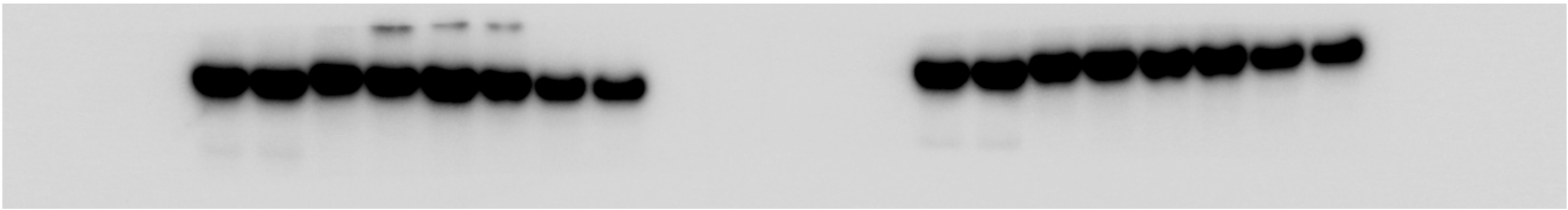

Supplement: Figure 2—source data 1. [file elife-101075-fig2-data1.zip › Figure 2-Source data 1/Fig 2H/Flag (CDKs).tif]

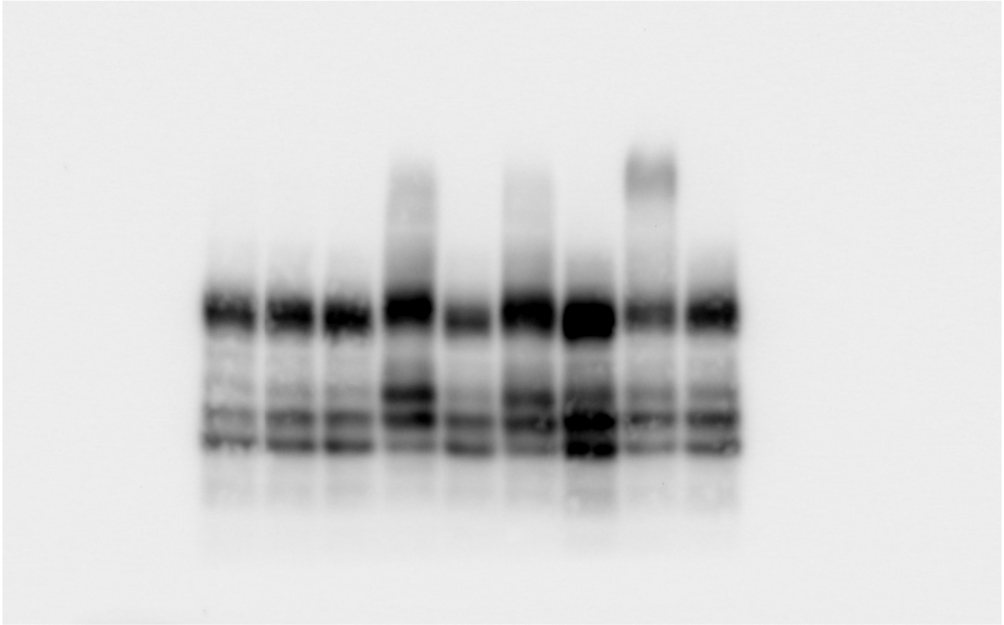

Supplement: Figure 2—source data 1. [file elife-101075-fig2-data1.zip › Figure 2-Source data 1/Fig 2H/HA (GTSE1) second.tif]

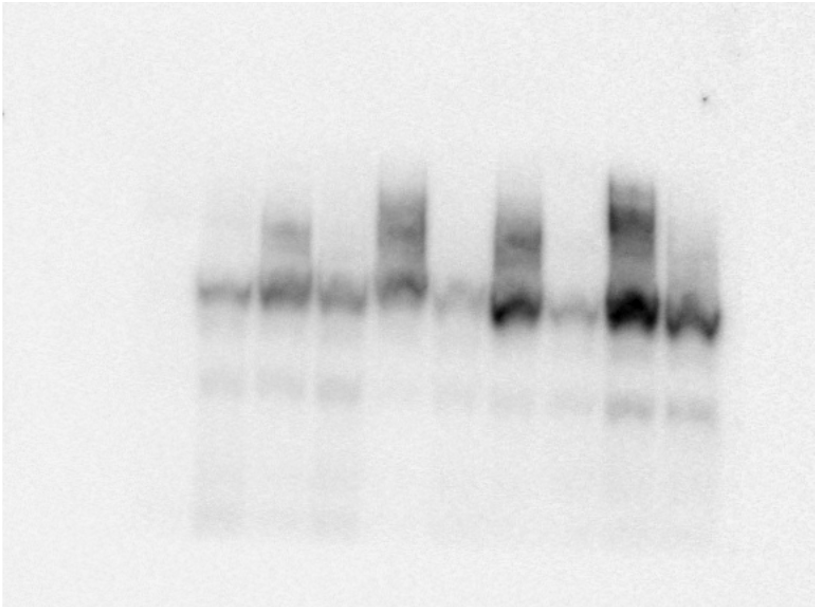

Supplement: Figure 2—source data 1. [file elife-101075-fig2-data1.zip › Figure 2-Source data 1/Fig 2H/HA (GTSE1) first.tif]

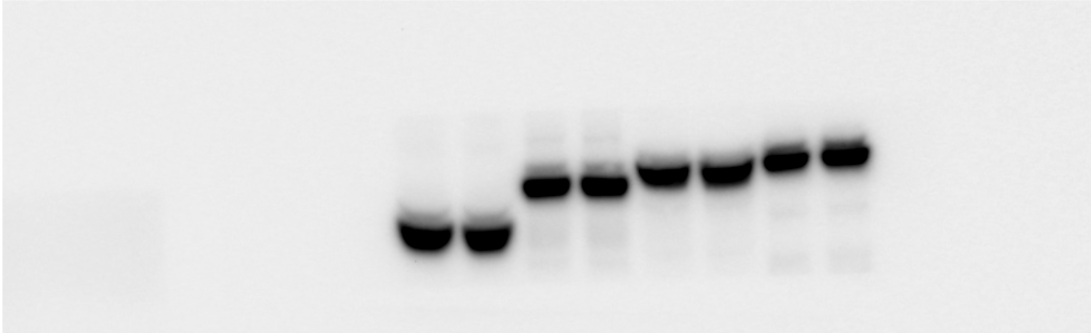

Supplement: Figure 2—source data 1. [file elife-101075-fig2-data1.zip › Figure 2-Source data 1/Fig 2H/GFP (Cyclins) second.tif]

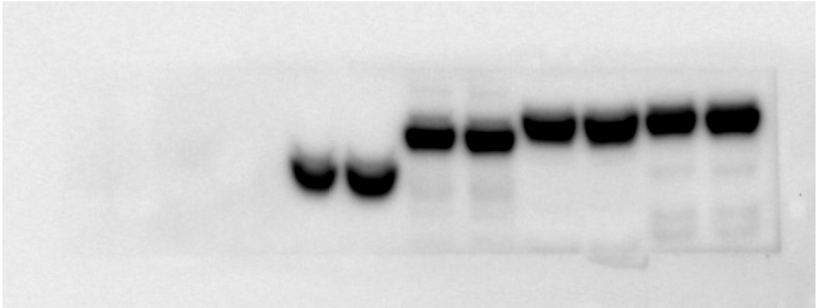

Supplement: Figure 2—source data 1. [file elife-101075-fig2-data1.zip › Figure 2-Source data 1/Fig 2H/GFP (Cyclins) first.tif]

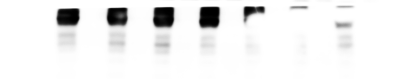

Supplement: Figure 2—source data 1. [file elife-101075-fig2-data1.zip › Figure 2-Source data 1/Fig 2F/Flag (CCND1 CDK4).tif]

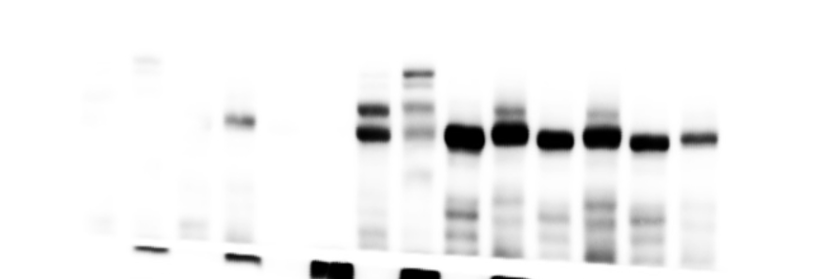

Supplement: Figure 2—source data 1. [file elife-101075-fig2-data1.zip › Figure 2-Source data 1/Fig 2F/HA (GTSE1) Short exposure.tif]

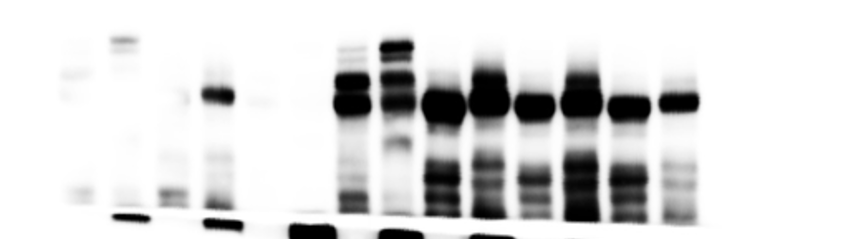

Supplement: Figure 2—source data 1. [file elife-101075-fig2-data1.zip › Figure 2-Source data 1/Fig 2F/HA (GTSE1) Long exposure.tif]

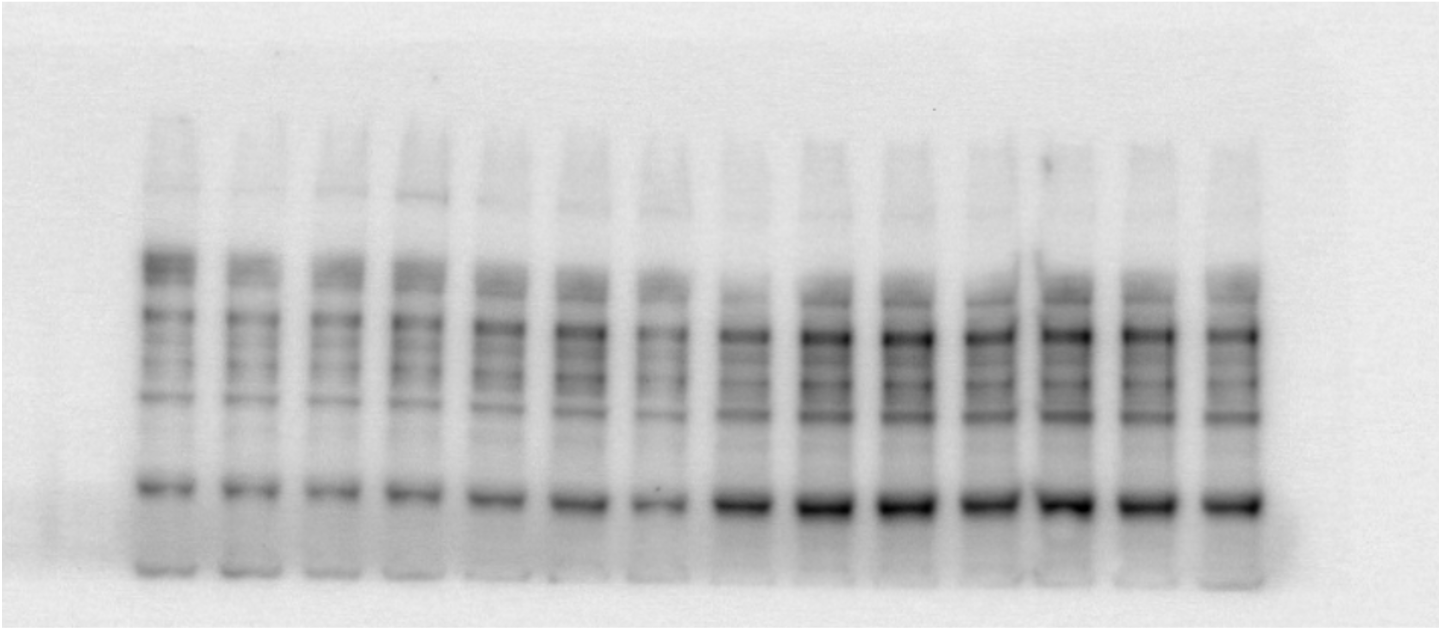

Supplement: Figure 2—source data 1. [file elife-101075-fig2-data1.zip › Figure 2-Source data 1/Fig 2G/GTSE1- p262.tif]

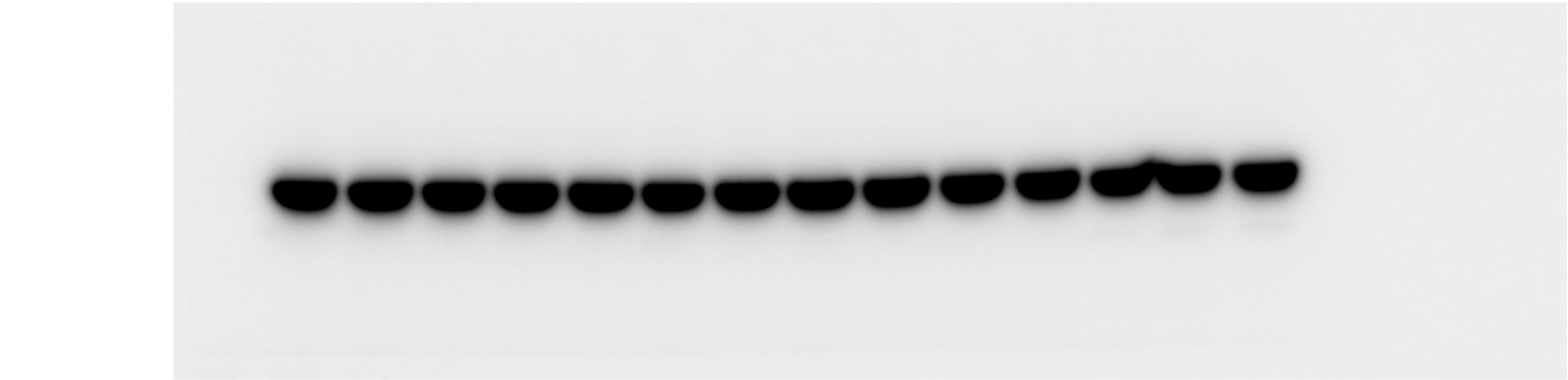

Supplement: Figure 2—source data 1. [file elife-101075-fig2-data1.zip › Figure 2-Source data 1/Fig 2G/Actin.tif]

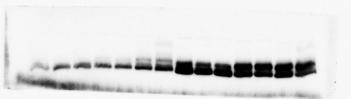

Supplement: Figure 2—source data 1. [file elife-101075-fig2-data1.zip › Figure 2-Source data 1/Fig 2G/GTSE1.tif]

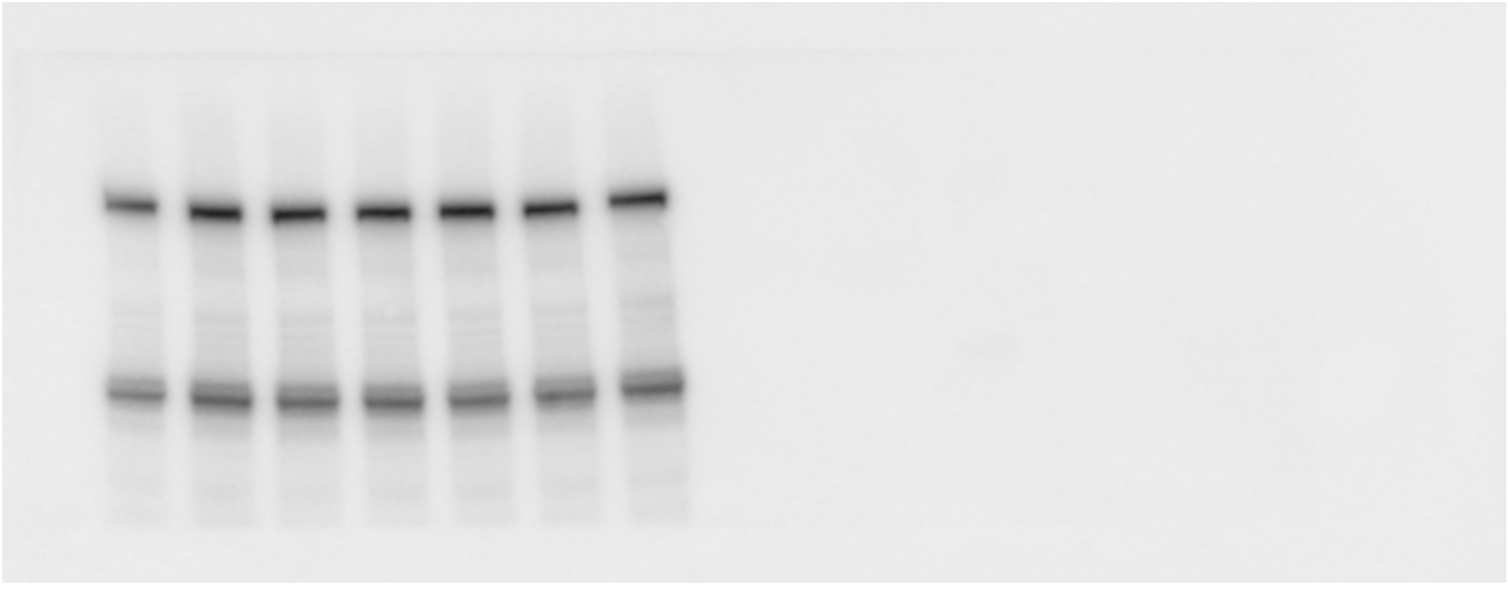

Supplement: Figure 2—source data 1. [file elife-101075-fig2-data1.zip › Figure 2-Source data 1/Fig 2G/AMBRA.tif]

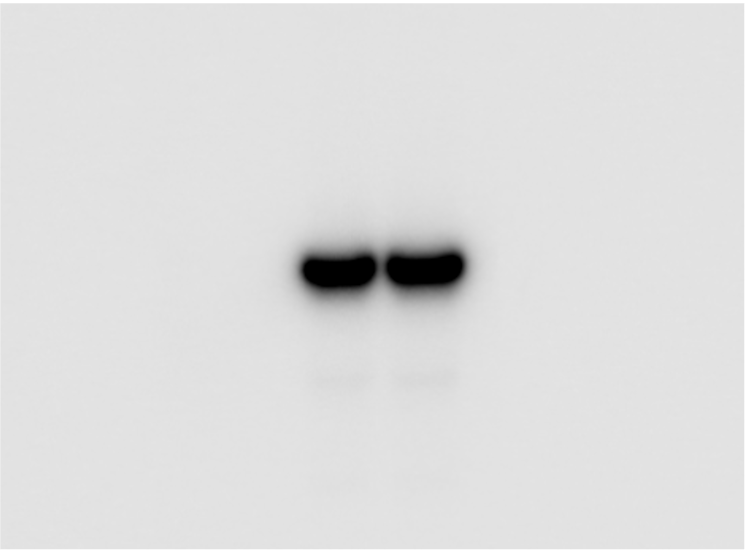

Supplement: Figure 2—source data 1. [file elife-101075-fig2-data1.zip › Figure 2-Source data 1/Fig 2B/cdk4.tif]

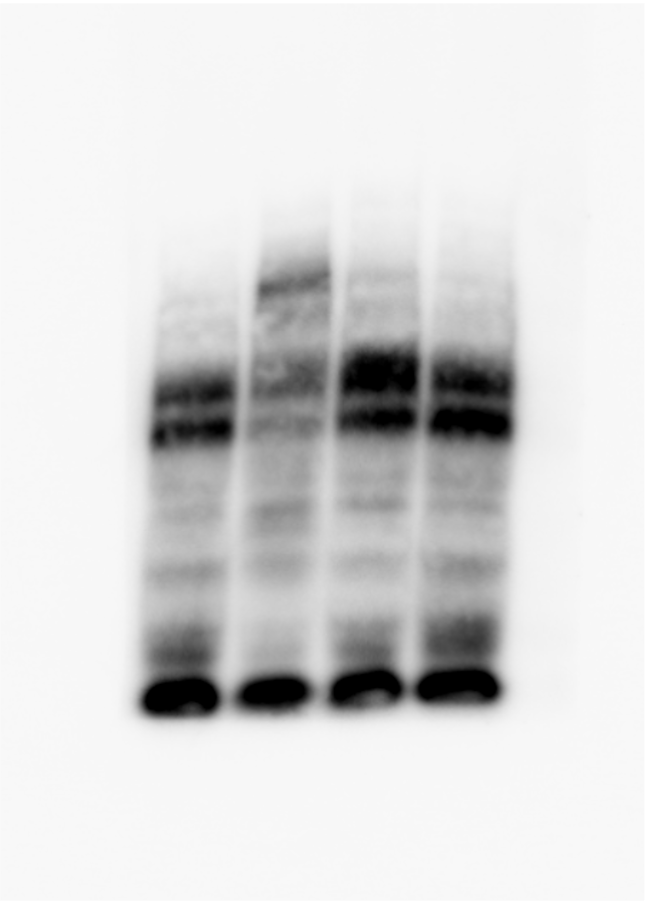

Supplement: Figure 2—source data 1. [file elife-101075-fig2-data1.zip › Figure 2-Source data 1/Fig 2B/gst.tif]

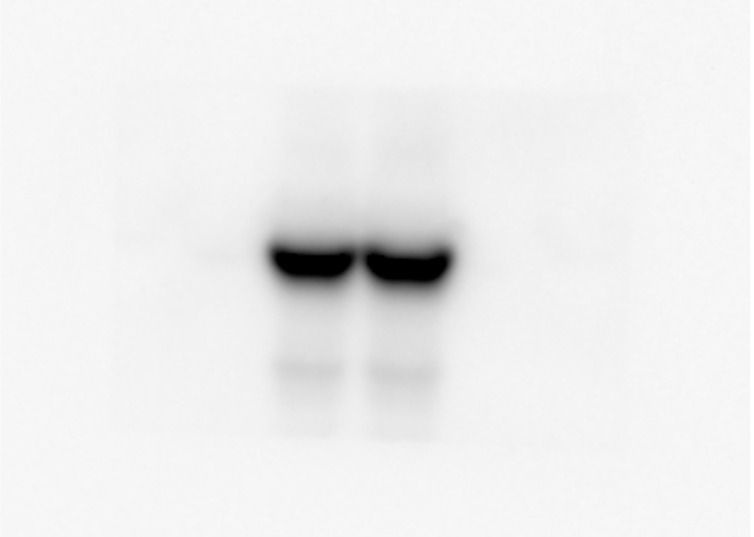

Supplement: Figure 2—source data 1. [file elife-101075-fig2-data1.zip › Figure 2-Source data 1/Fig 2B/ccnd1.tif]

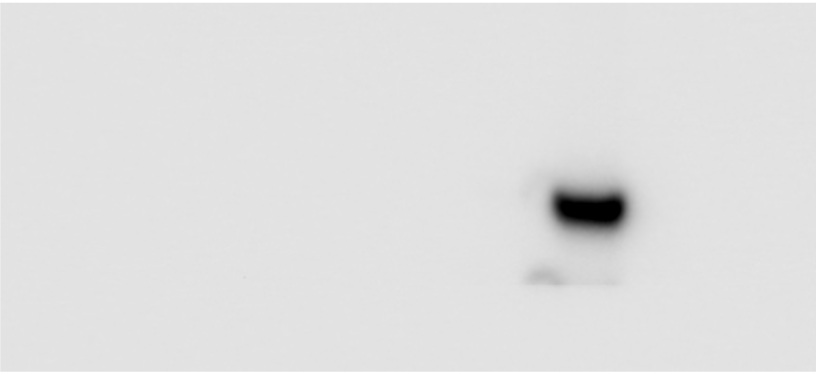

Supplement: Figure 2—source data 1. [file elife-101075-fig2-data1.zip › Figure 2-Source data 1/Fig 2B/ERK2.tif]

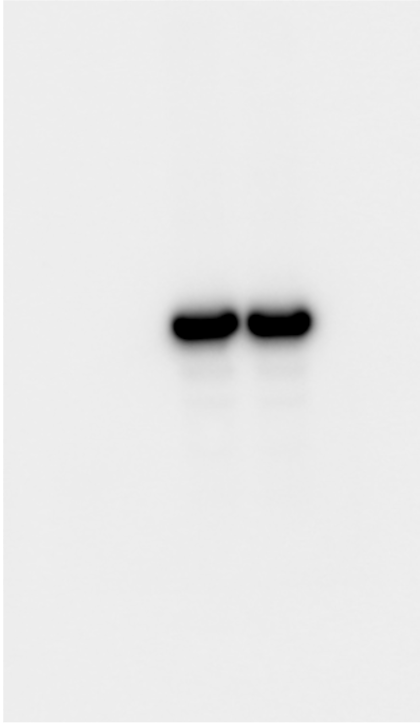

Supplement: Figure 2—source data 1. [file elife-101075-fig2-data1.zip › Figure 2-Source data 1/Fig 2C/CDK4.tif]

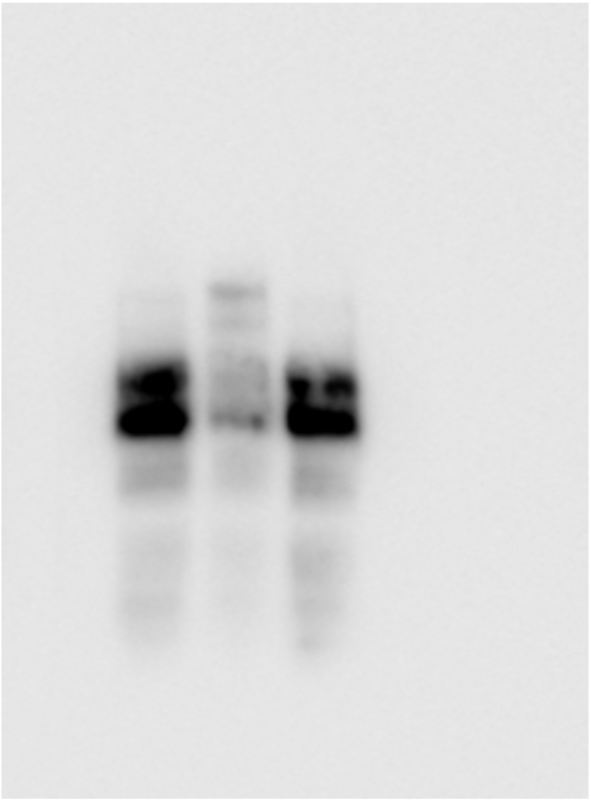

Supplement: Figure 2—source data 1. [file elife-101075-fig2-data1.zip › Figure 2-Source data 1/Fig 2C/His (Rb).tif]

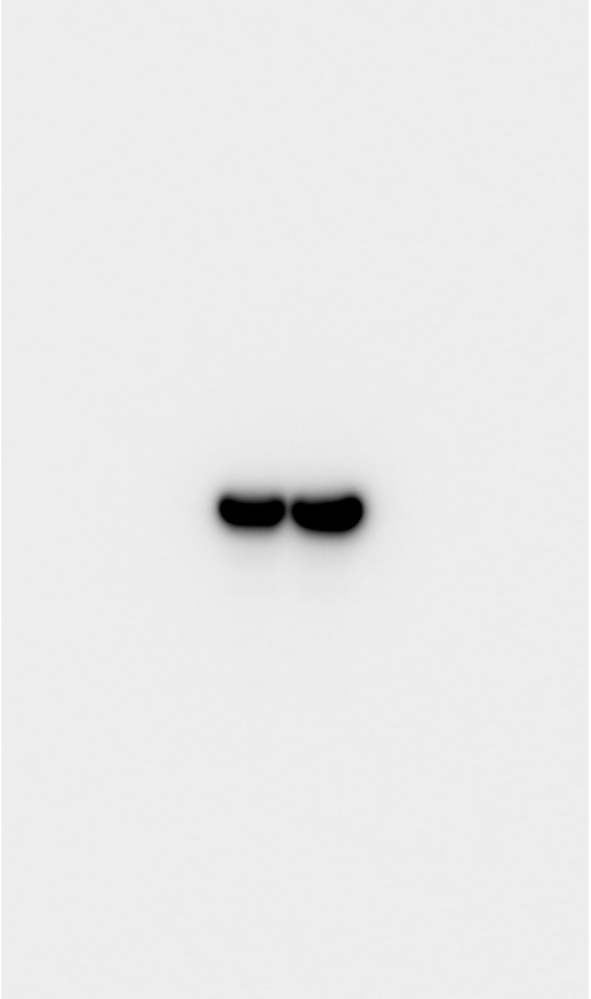

Supplement: Figure 2—source data 1. [file elife-101075-fig2-data1.zip › Figure 2-Source data 1/Fig 2C/CCND1.tif]

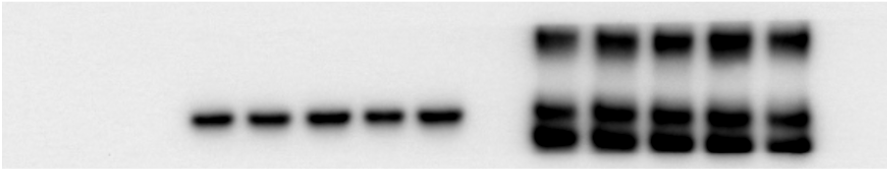

Supplement: Figure 2—source data 1. [file elife-101075-fig2-data1.zip › Figure 2-Source data 1/Fig 2D/Tubulin.tif]

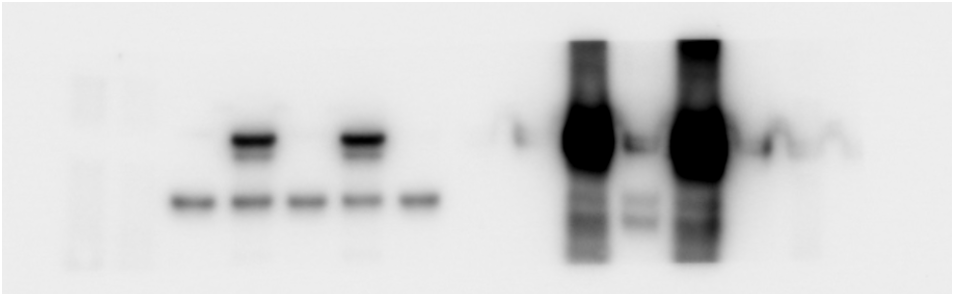

Supplement: Figure 2—source data 1. [file elife-101075-fig2-data1.zip › Figure 2-Source data 1/Fig 2D/CDK4.tif]

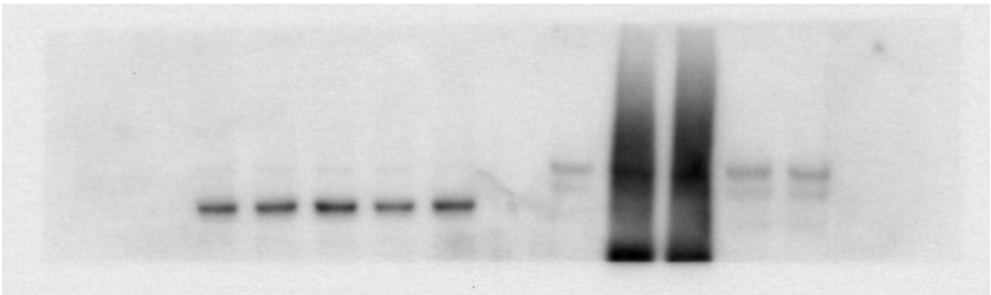

Supplement: Figure 2—source data 1. [file elife-101075-fig2-data1.zip › Figure 2-Source data 1/Fig 2D/pRb.tif]

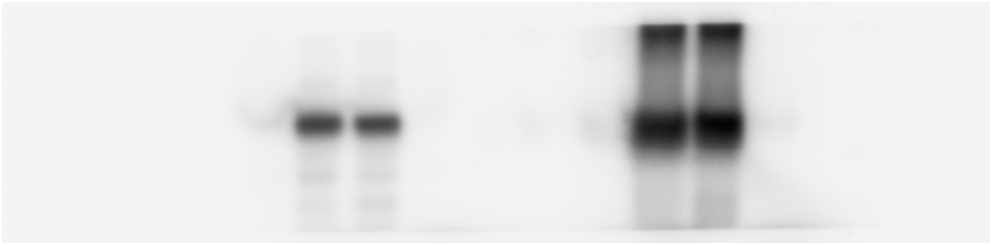

Supplement: Figure 2—source data 1. [file elife-101075-fig2-data1.zip › Figure 2-Source data 1/Fig 2D/CCND1.tif]

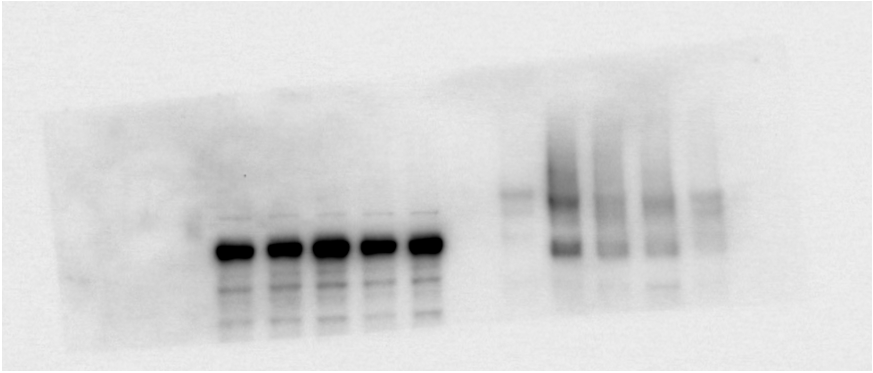

Supplement: Figure 2—source data 1. [file elife-101075-fig2-data1.zip › Figure 2-Source data 1/Fig 2D/GTSE1.tif]

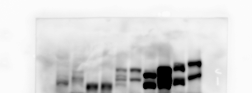

Supplement: Figure 2—source data 1. [file elife-101075-fig2-data1.zip › Figure 2-Source data 1/Fig2A/Flag.tif]

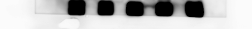

Supplement: Figure 2—source data 1. [file elife-101075-fig2-data1.zip › Figure 2-Source data 1/Fig2A/CCND1.tif]

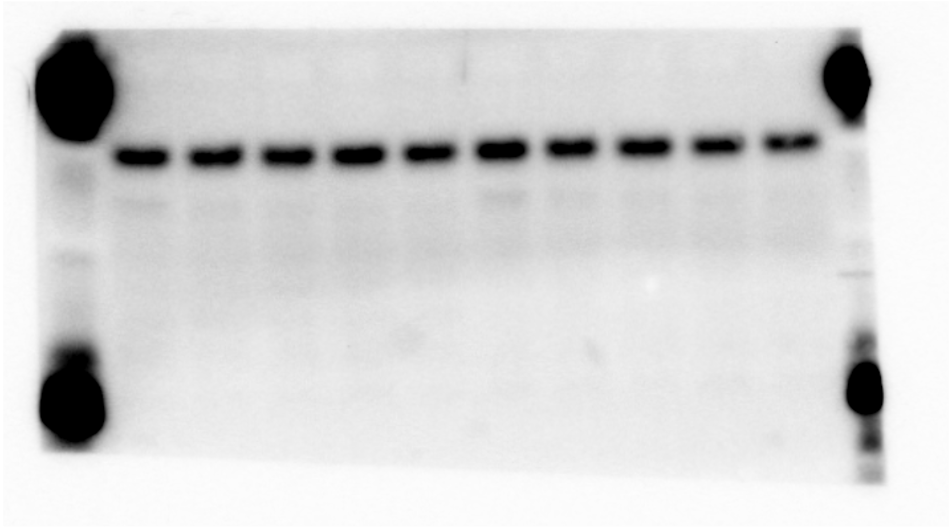

Supplement: Figure 3—source data 1. [file elife-101075-fig3-data1.zip › Figure 3-Source data 1/Fig 3C/SKP1.tif]

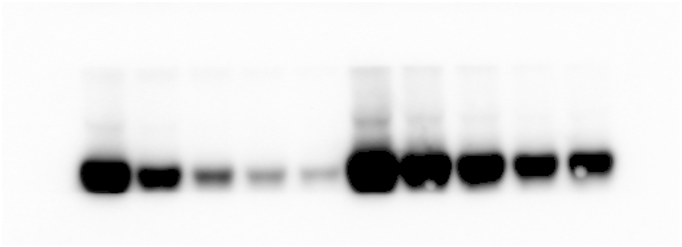

Supplement: Figure 3—source data 1. [file elife-101075-fig3-data1.zip › Figure 3-Source data 1/Fig 3C/CCND1.tif]

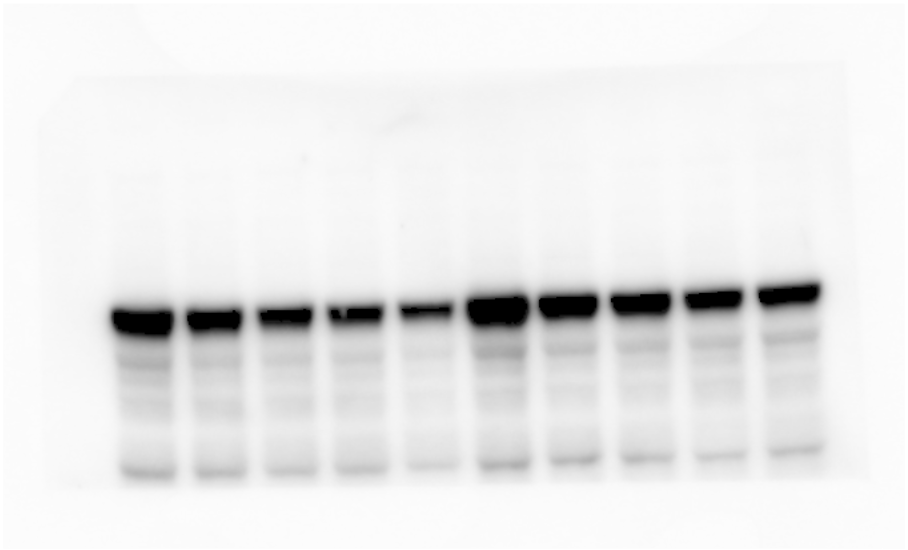

Supplement: Figure 3—source data 1. [file elife-101075-fig3-data1.zip › Figure 3-Source data 1/Fig 3C/GTSE1.tif]

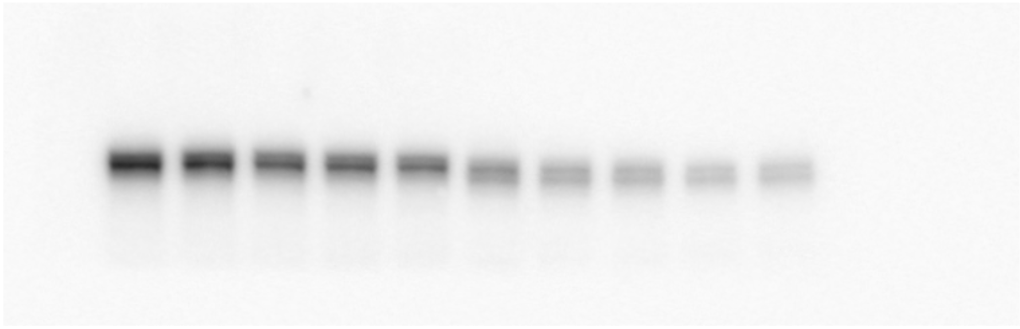

Supplement: Figure 3—source data 1. [file elife-101075-fig3-data1.zip › Figure 3-Source data 1/Fig 3C/AMBRA1.tif]

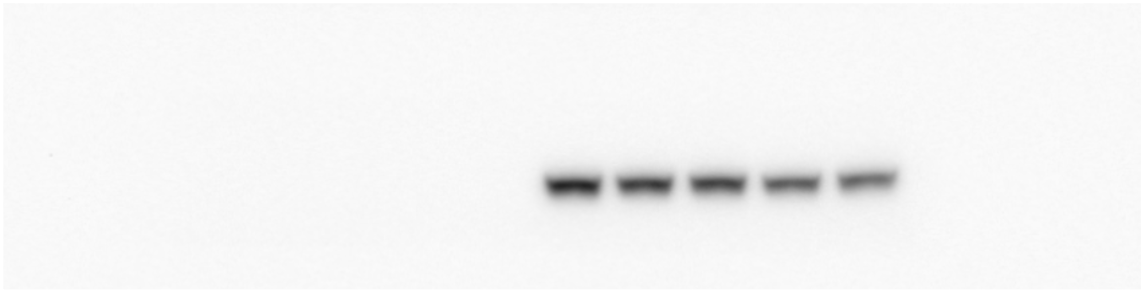

Supplement: Figure 3—source data 1. [file elife-101075-fig3-data1.zip › Figure 3-Source data 1/Fig 3C/HA (TIR1).tif]

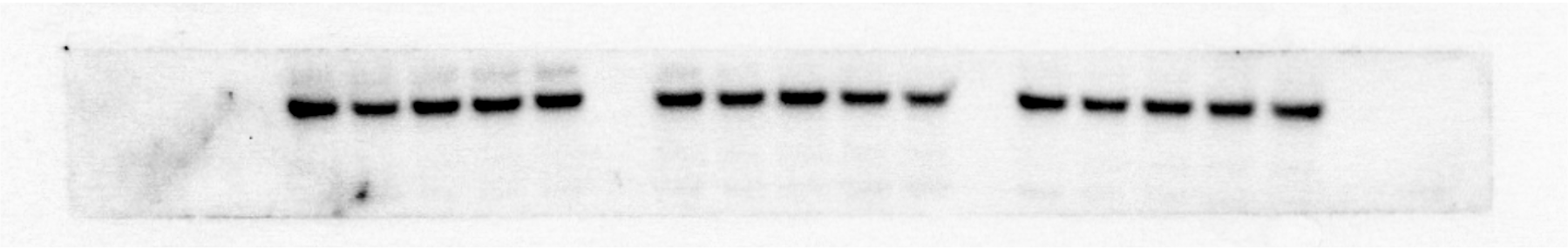

Supplement: Figure 3—source data 1. [file elife-101075-fig3-data1.zip › Figure 3-Source data 1/Fig 3D/Tubulin.tif]

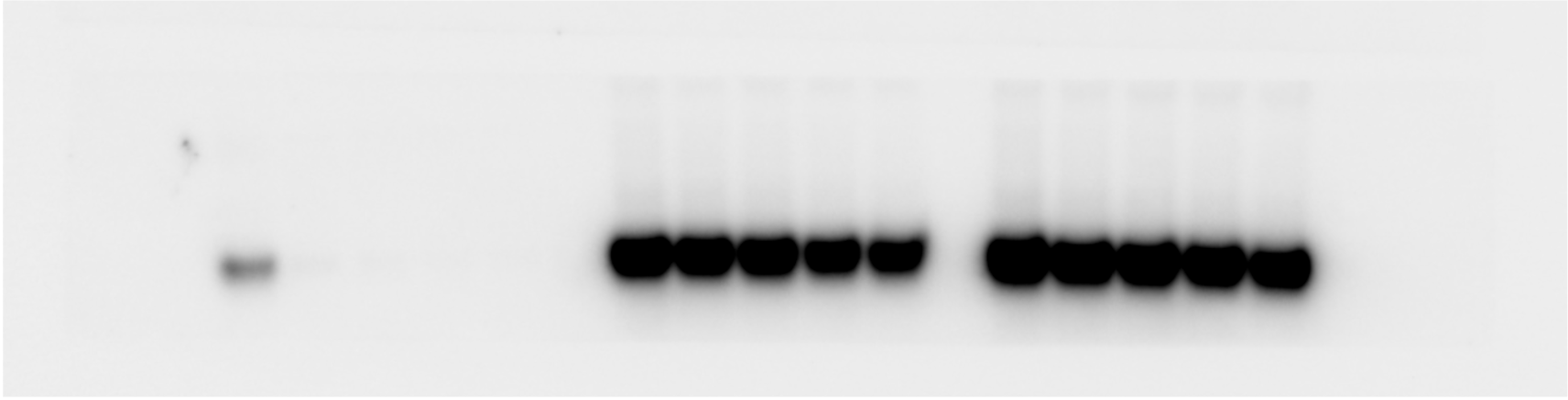

Supplement: Figure 3—source data 1. [file elife-101075-fig3-data1.zip › Figure 3-Source data 1/Fig 3D/CCND1.tif]

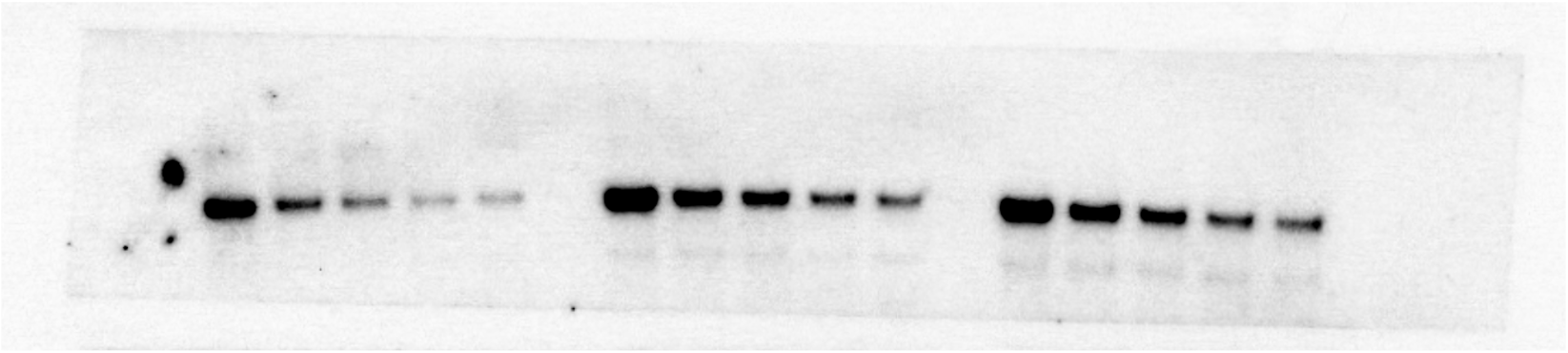

Supplement: Figure 3—source data 1. [file elife-101075-fig3-data1.zip › Figure 3-Source data 1/Fig 3D/GTSE1.tif]

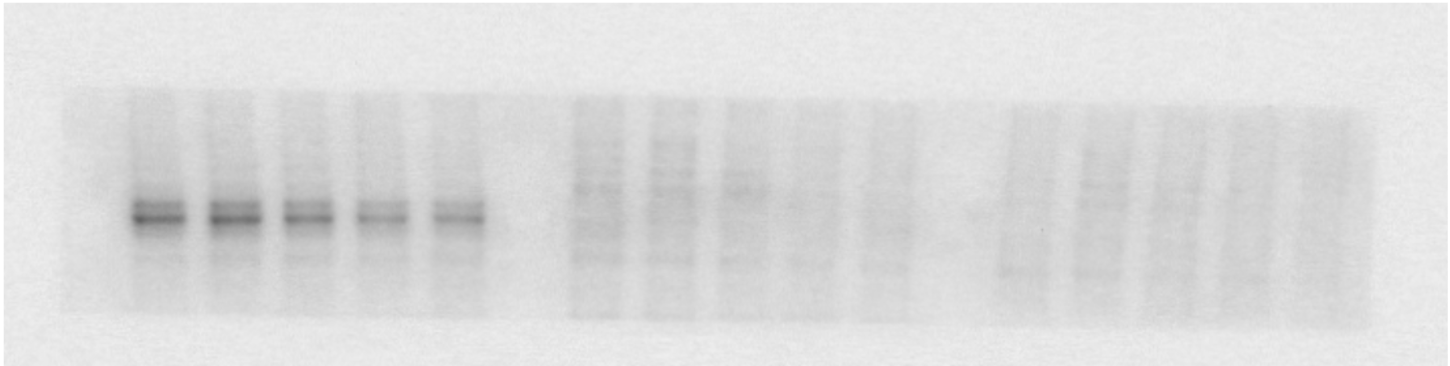

Supplement: Figure 3—source data 1. [file elife-101075-fig3-data1.zip › Figure 3-Source data 1/Fig 3D/AMBRA1.tif]

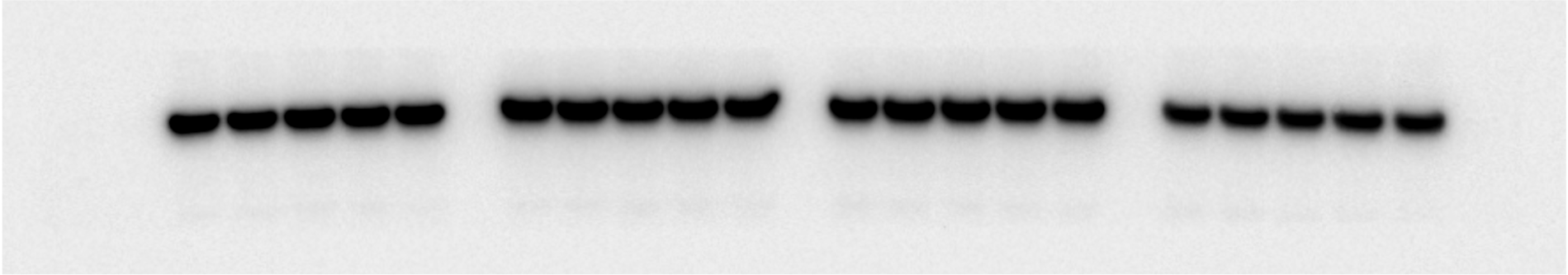

Supplement: Figure 3—source data 1. [file elife-101075-fig3-data1.zip › Figure 3-Source data 1/Fig 3E/Tubulin.tif]

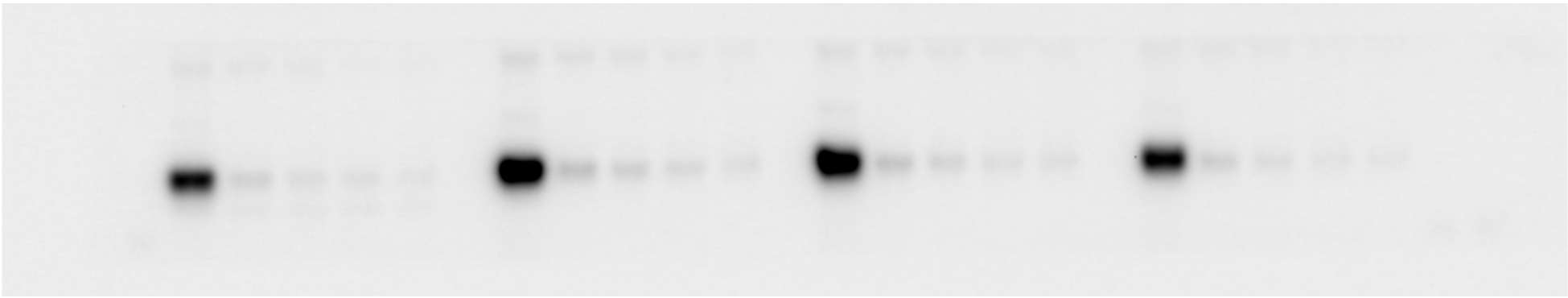

Supplement: Figure 3—source data 1. [file elife-101075-fig3-data1.zip › Figure 3-Source data 1/Fig 3E/CCND1.tif]

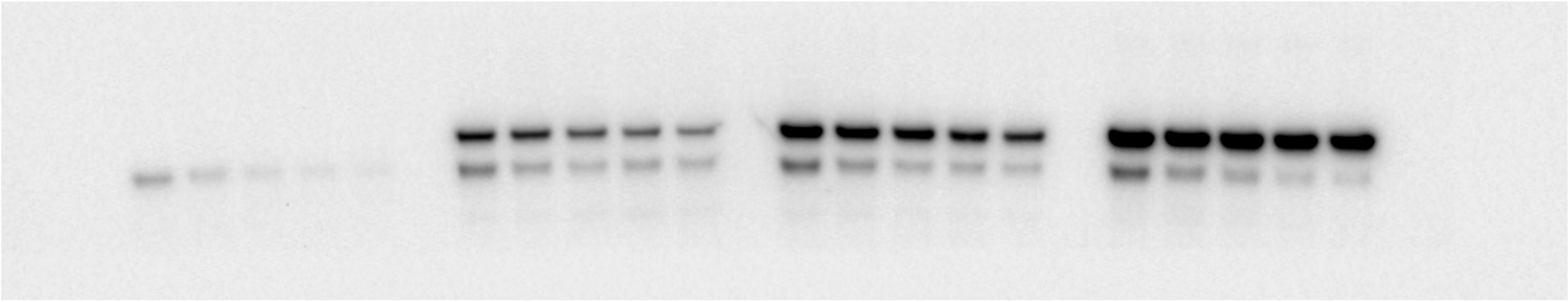

Supplement: Figure 3—source data 1. [file elife-101075-fig3-data1.zip › Figure 3-Source data 1/Fig 3E/GTSE1.tif]

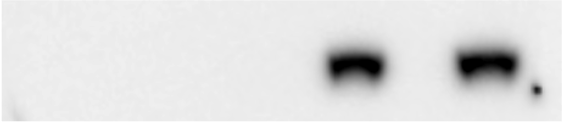

Supplement: Figure 3—source data 1. [file elife-101075-fig3-data1.zip › Figure 3-Source data 1/Fig 3B/HA (Os-Tir1).tif]

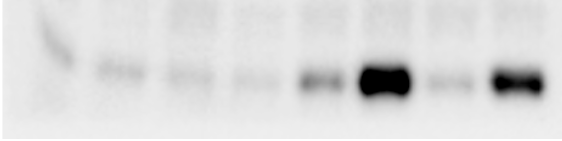

Supplement: Figure 3—source data 1. [file elife-101075-fig3-data1.zip › Figure 3-Source data 1/Fig 3B/CCND3.tif]

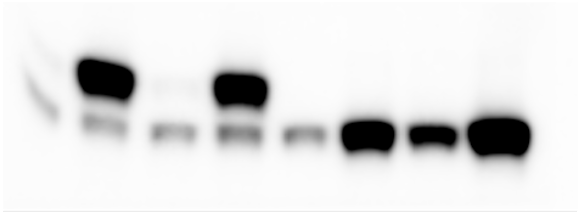

Supplement: Figure 3—source data 1. [file elife-101075-fig3-data1.zip › Figure 3-Source data 1/Fig 3B/CCND1.tif]

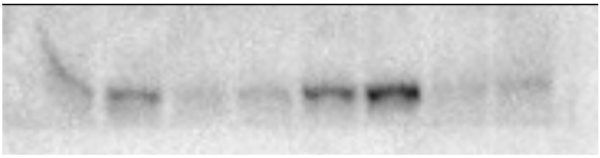

Supplement: Figure 3—source data 1. [file elife-101075-fig3-data1.zip › Figure 3-Source data 1/Fig 3B/GTSE1.tif]

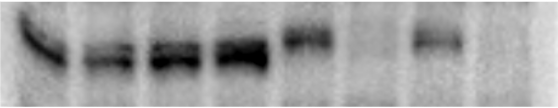

Supplement: Figure 3—source data 1. [file elife-101075-fig3-data1.zip › Figure 3-Source data 1/Fig 3B/AMBRA1.tif]

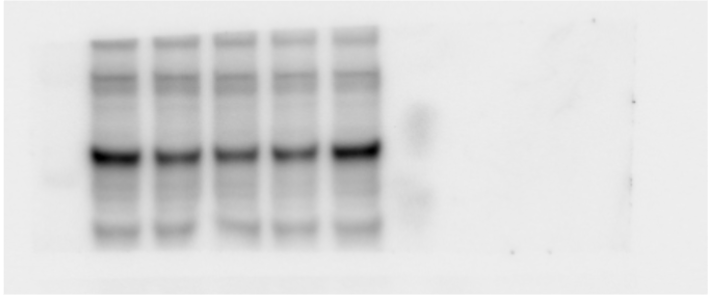

Supplement: Figure 3—source data 1. [file elife-101075-fig3-data1.zip › Figure 3-Source data 1/Fig 3I/P62.tif]

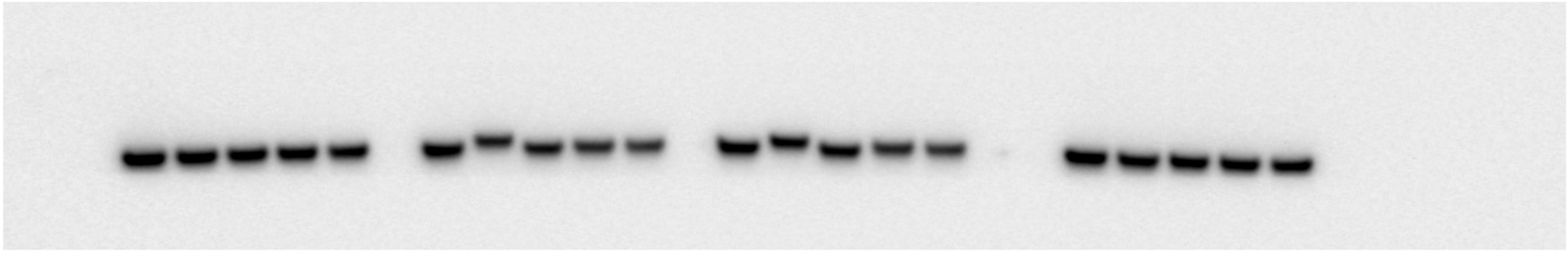

Supplement: Figure 3—source data 1. [file elife-101075-fig3-data1.zip › Figure 3-Source data 1/Fig 3I/Tubulin.tif]

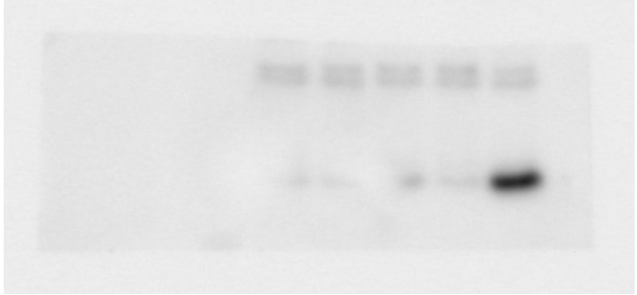

Supplement: Figure 3—source data 1. [file elife-101075-fig3-data1.zip › Figure 3-Source data 1/Fig 3I/LC3.tif]

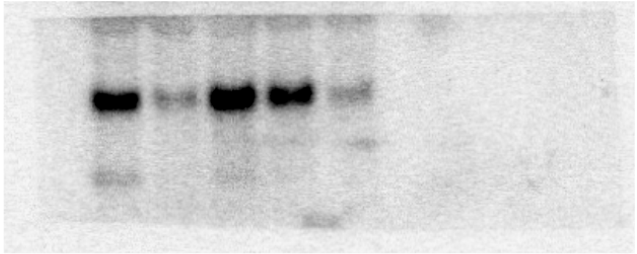

Supplement: Figure 3—source data 1. [file elife-101075-fig3-data1.zip › Figure 3-Source data 1/Fig 3I/P27.tif]

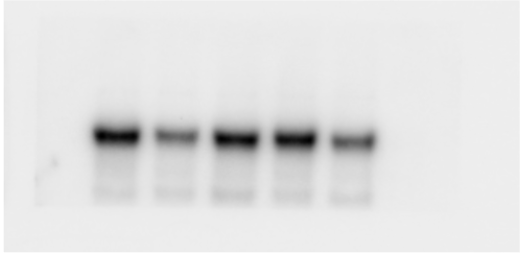

Supplement: Figure 3—source data 1. [file elife-101075-fig3-data1.zip › Figure 3-Source data 1/Fig 3I/GTSE1.tif]

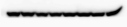

Supplement: Figure 3—source data 1. [file elife-101075-fig3-data1.zip › Figure 3-Source data 1/Fig 3A/Tubulin.tif]

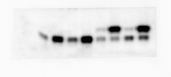

Supplement: Figure 3—source data 1. [file elife-101075-fig3-data1.zip › Figure 3-Source data 1/Fig 3A/CCND1.tif]

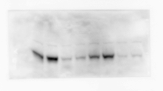

Supplement: Figure 3—source data 1. [file elife-101075-fig3-data1.zip › Figure 3-Source data 1/Fig 3A/GTSE1.tif]

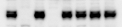

Supplement: Figure 3—source data 1. [file elife-101075-fig3-data1.zip › Figure 3-Source data 1/Fig 3A/AMBRA1.tif]

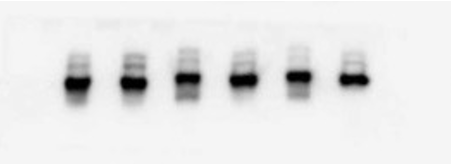

Supplement: Figure 3—figure supplement 1—source data 1. [file elife-101075-fig3-figsupp1-data1.zip › Figure 3-figure supplement 1- Source data 1/Figure 3- figure supplement 1A/CCND1 second panel.tif]

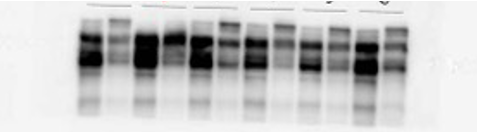

Supplement: Figure 3—figure supplement 1—source data 1. [file elife-101075-fig3-figsupp1-data1.zip › Figure 3-figure supplement 1- Source data 1/Figure 3- figure supplement 1A/GTSE1 First panel.tif]

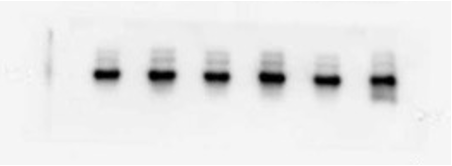

Supplement: Figure 3—figure supplement 1—source data 1. [file elife-101075-fig3-figsupp1-data1.zip › Figure 3-figure supplement 1- Source data 1/Figure 3- figure supplement 1A/CCND1 first panel.tif]

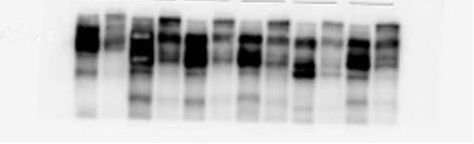

Supplement: Figure 3—figure supplement 1—source data 1. [file elife-101075-fig3-figsupp1-data1.zip › Figure 3-figure supplement 1- Source data 1/Figure 3- figure supplement 1A/GTSE1 second panel.tif]

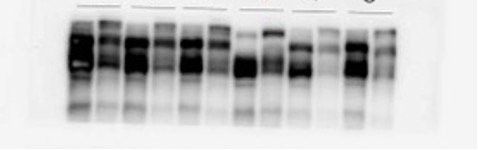

Supplement: Figure 3—figure supplement 1—source data 1. [file elife-101075-fig3-figsupp1-data1.zip › Figure 3-figure supplement 1- Source data 1/Figure 3- figure supplement 1A/GTSE1 third panel.tif]

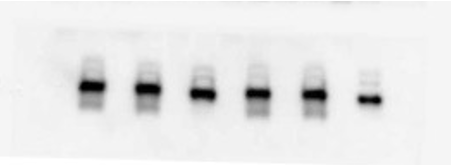

Supplement: Figure 3—figure supplement 1—source data 1. [file elife-101075-fig3-figsupp1-data1.zip › Figure 3-figure supplement 1- Source data 1/Figure 3- figure supplement 1A/CCND1 third panel.tif]

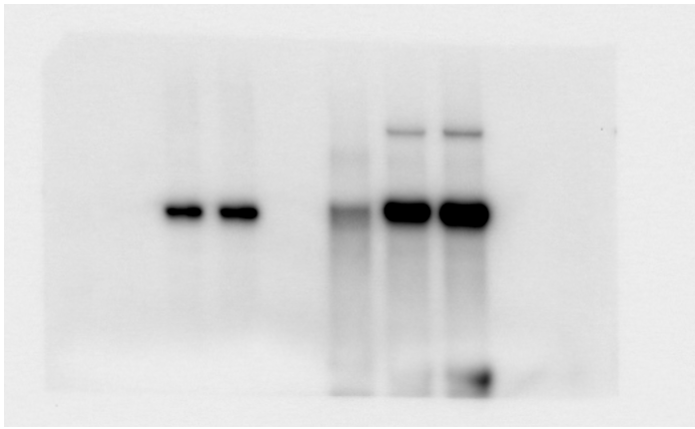

Supplement: Figure 3—figure supplement 1—source data 1. [file elife-101075-fig3-figsupp1-data1.zip › Figure 3-figure supplement 1- Source data 1/Figure 3- figure supplement 1E/DDB1.tif]

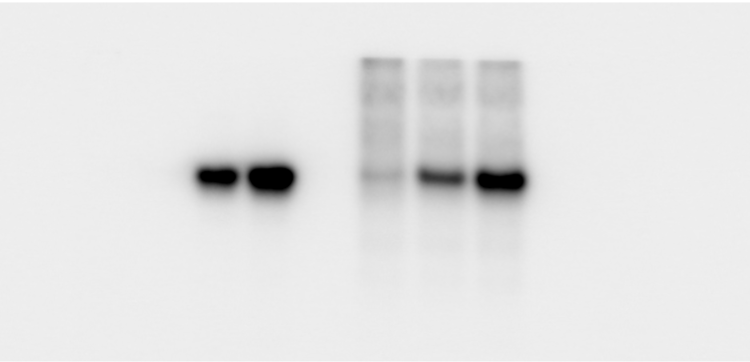

Supplement: Figure 3—figure supplement 1—source data 1. [file elife-101075-fig3-figsupp1-data1.zip › Figure 3-figure supplement 1- Source data 1/Figure 3- figure supplement 1E/CCND1.tif]

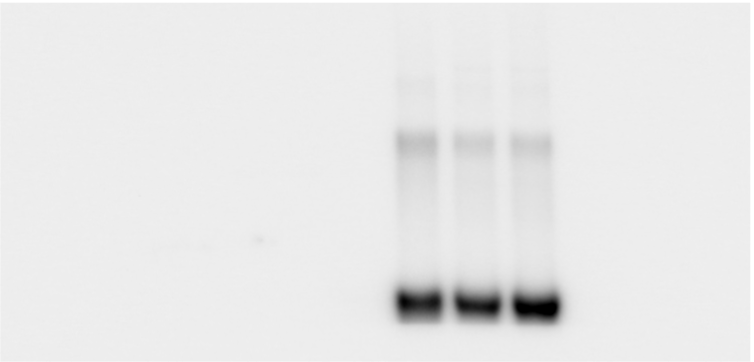

Supplement: Figure 3—figure supplement 1—source data 1. [file elife-101075-fig3-figsupp1-data1.zip › Figure 3-figure supplement 1- Source data 1/Figure 3- figure supplement 1E/GTSE1.tif]

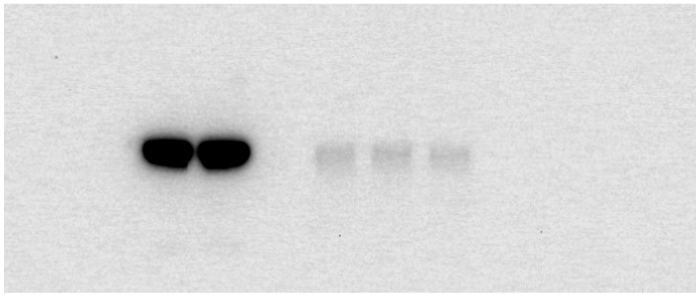

Supplement: Figure 3—figure supplement 1—source data 1. [file elife-101075-fig3-figsupp1-data1.zip › Figure 3-figure supplement 1- Source data 1/Figure 3- figure supplement 1E/Vinculin.tif]

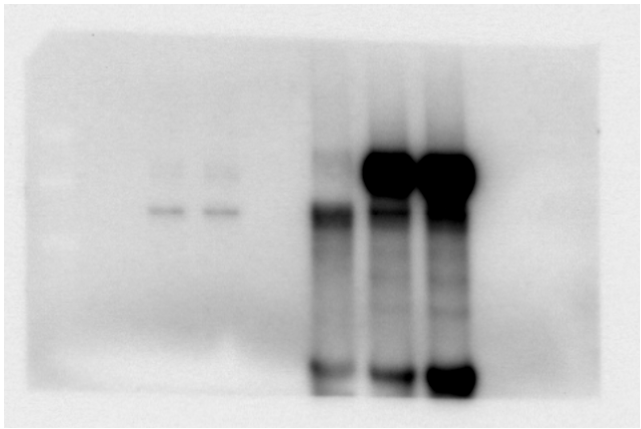

Supplement: Figure 3—figure supplement 1—source data 1. [file elife-101075-fig3-figsupp1-data1.zip › Figure 3-figure supplement 1- Source data 1/Figure 3- figure supplement 1E/AMBRA1.tif]

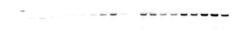

Supplement: Figure 3—figure supplement 1—source data 1. [file elife-101075-fig3-figsupp1-data1.zip › Figure 3-figure supplement 1- Source data 1/Figure 3- figure supplement 1B/CCNA2.tif]

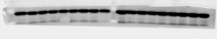

Supplement: Figure 3—figure supplement 1—source data 1. [file elife-101075-fig3-figsupp1-data1.zip › Figure 3-figure supplement 1- Source data 1/Figure 3- figure supplement 1B/PCNA.tif]

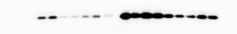

Supplement: Figure 3—figure supplement 1—source data 1. [file elife-101075-fig3-figsupp1-data1.zip › Figure 3-figure supplement 1- Source data 1/Figure 3- figure supplement 1B/P21.tif]

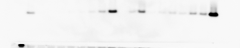

Supplement: Figure 3—figure supplement 1—source data 1. [file elife-101075-fig3-figsupp1-data1.zip › Figure 3-figure supplement 1- Source data 1/Figure 3- figure supplement 1B/pH3 (S10).tif]

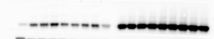

Supplement: Figure 3—figure supplement 1—source data 1. [file elife-101075-fig3-figsupp1-data1.zip › Figure 3-figure supplement 1- Source data 1/Figure 3- figure supplement 1B/CCND1.tif]

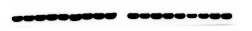

Supplement: Figure 3—figure supplement 1—source data 1. [file elife-101075-fig3-figsupp1-data1.zip › Figure 3-figure supplement 1- Source data 1/Figure 3- figure supplement 1B/GAPDH.tif]

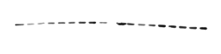

Supplement: Figure 3—figure supplement 1—source data 1. [file elife-101075-fig3-figsupp1-data1.zip › Figure 3-figure supplement 1- Source data 1/Figure 3- figure supplement 1B/GTSE1.tif]

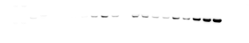

Supplement: Figure 3—figure supplement 1—source data 1. [file elife-101075-fig3-figsupp1-data1.zip › Figure 3-figure supplement 1- Source data 1/Figure 3- figure supplement 1B/CCNB1.tif]

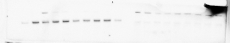

Supplement: Figure 3—figure supplement 1—source data 1. [file elife-101075-fig3-figsupp1-data1.zip › Figure 3-figure supplement 1- Source data 1/Figure 3- figure supplement 1B/AMBRA1.tif]

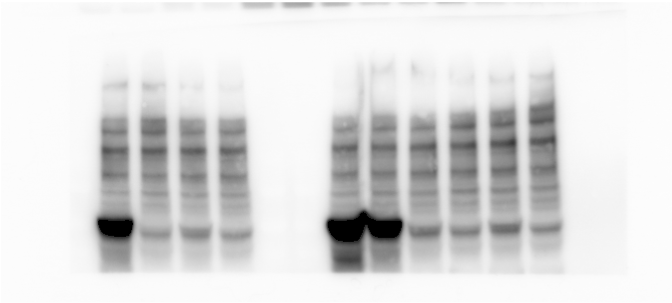

Supplement: Figure 3—figure supplement 1—source data 1. [file elife-101075-fig3-figsupp1-data1.zip › Figure 3-figure supplement 1- Source data 1/Figure 3- figure supplement 1C/pGTSE1 (S262).tif]

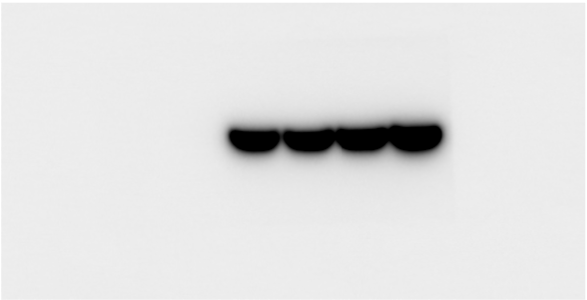

Supplement: Figure 3—figure supplement 1—source data 1. [file elife-101075-fig3-figsupp1-data1.zip › Figure 3-figure supplement 1- Source data 1/Figure 3- figure supplement 1C/Actin.tif]

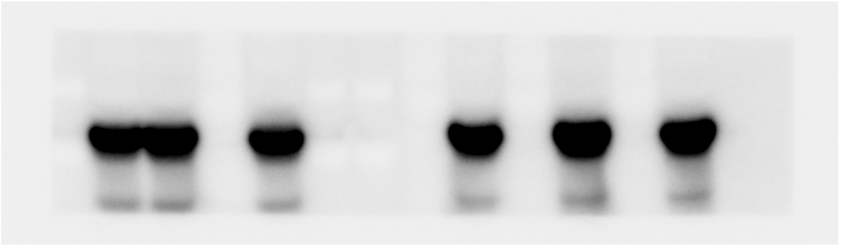

Supplement: Figure 3—figure supplement 1—source data 1. [file elife-101075-fig3-figsupp1-data1.zip › Figure 3-figure supplement 1- Source data 1/Figure 3- figure supplement 1C/Flag (ccnd1).tif]

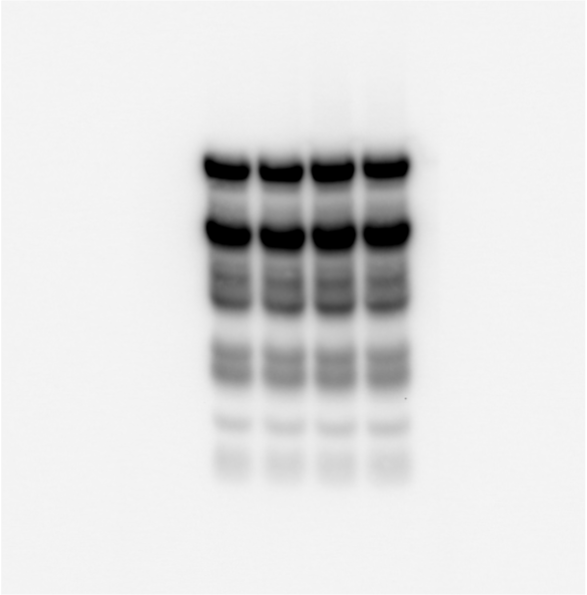

Supplement: Figure 3—figure supplement 1—source data 1. [file elife-101075-fig3-figsupp1-data1.zip › Figure 3-figure supplement 1- Source data 1/Figure 3- figure supplement 1C/HA (GTSE1).tif]
