# Supplementary material for: Stabilization of GTSE1 by cyclin D1–CDK4/6-mediated phosphorylation promotes cell proliferation with implications for cancer prognosis
Source: eLife. 2025 Apr 24;13:RP101075. doi: 10.7554/eLife.101075 (PMC12021411; doi:10.7554/eLife.101075)
Supplement: Figure 2—source data 2. [file elife-101075-fig2-data2.pdf]

Figure 2A

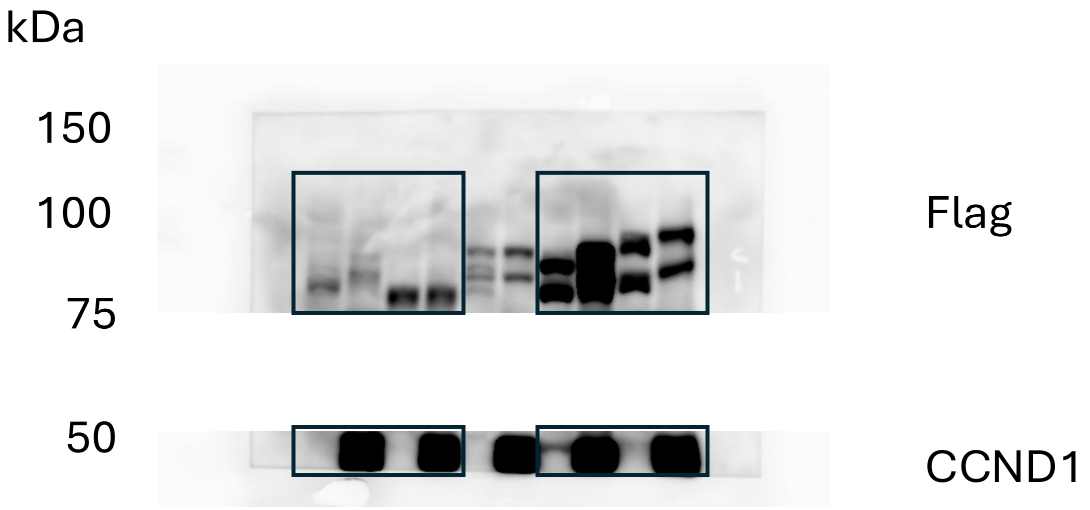

Figure 2B

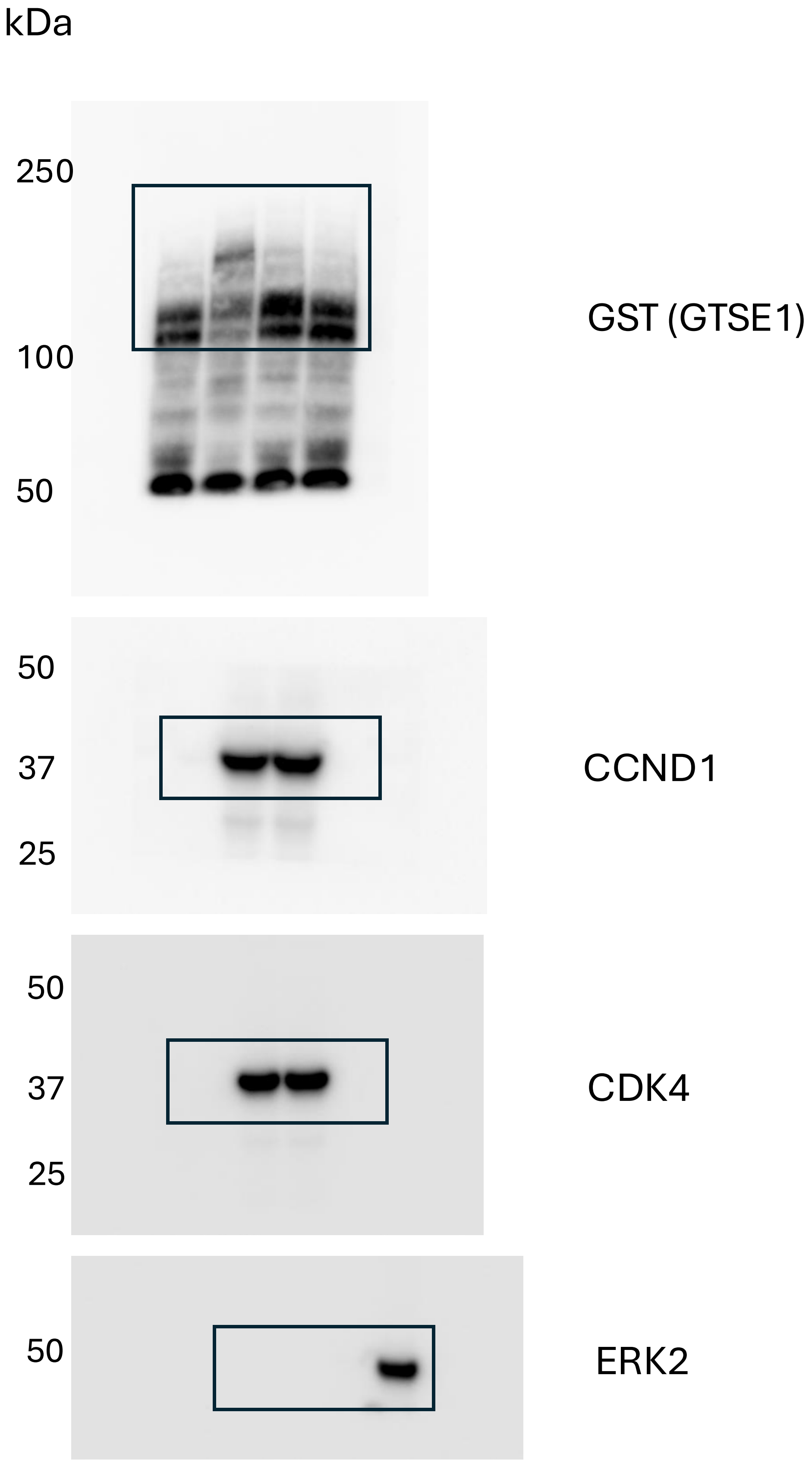

Figure 2C

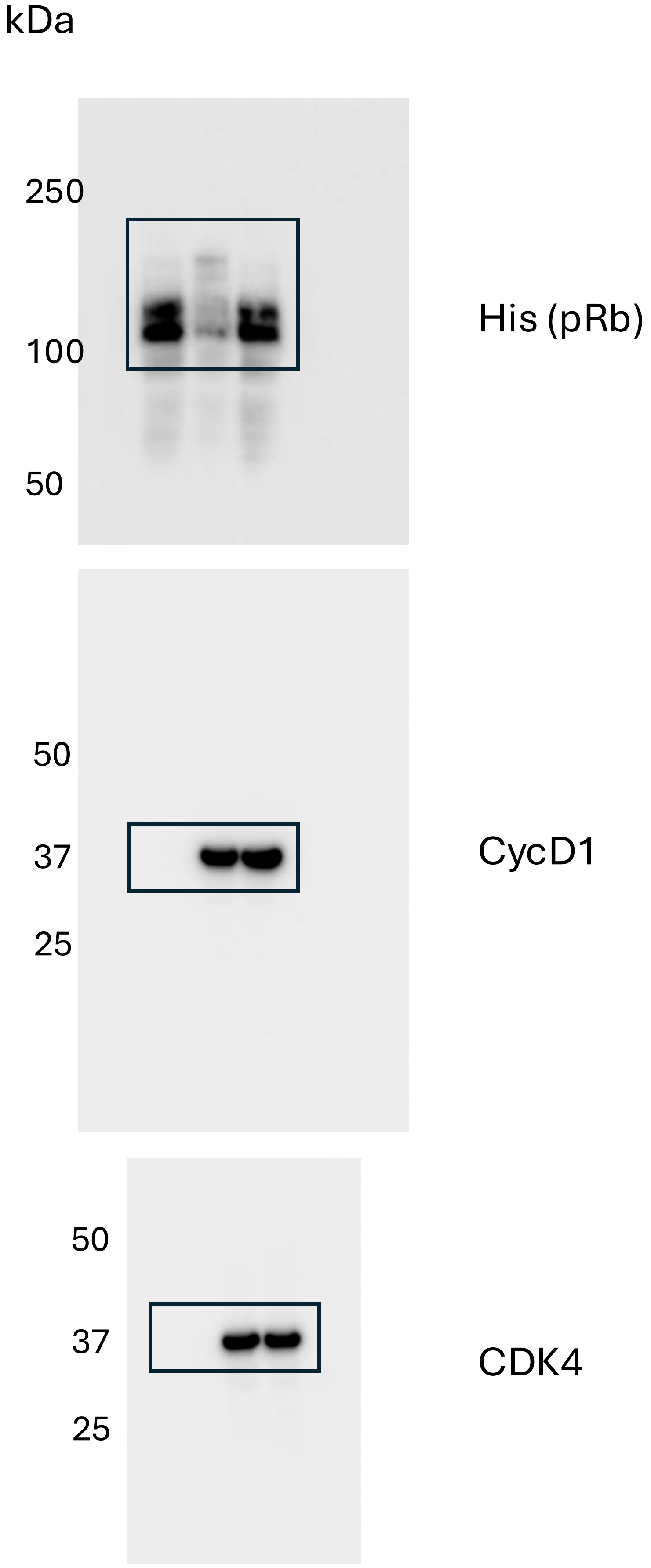

Figure 2D

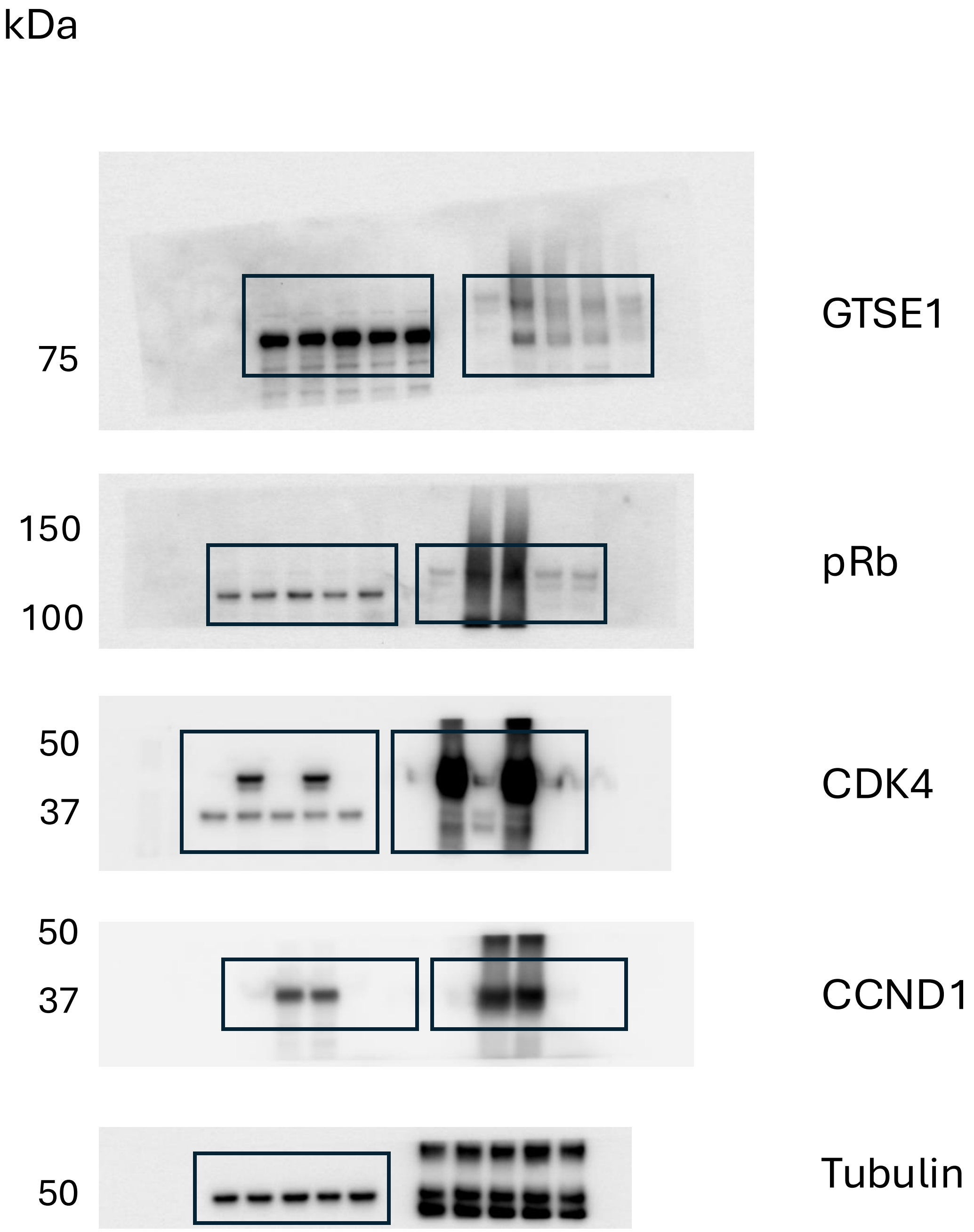

Figure 2F

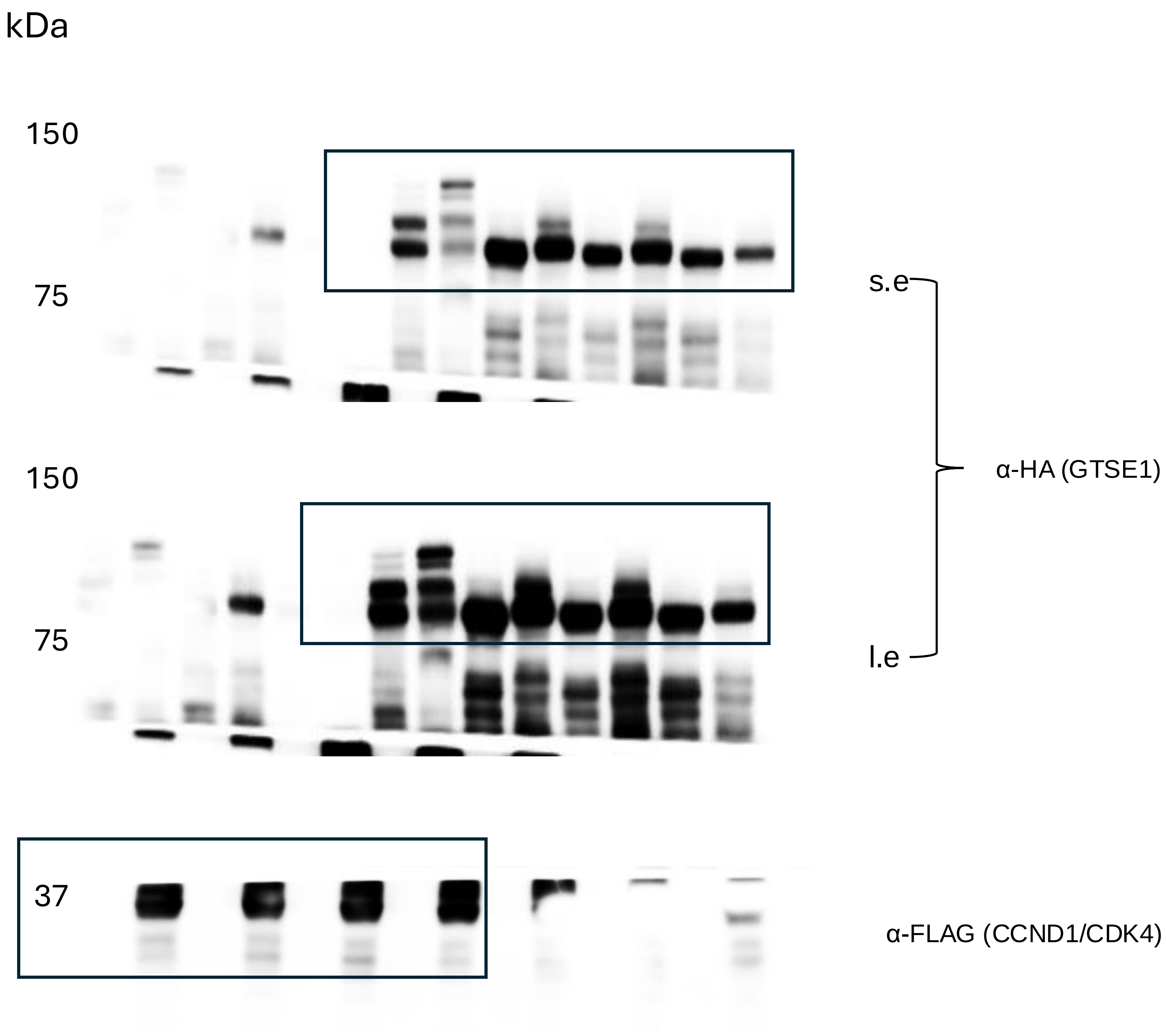

Figure 2G

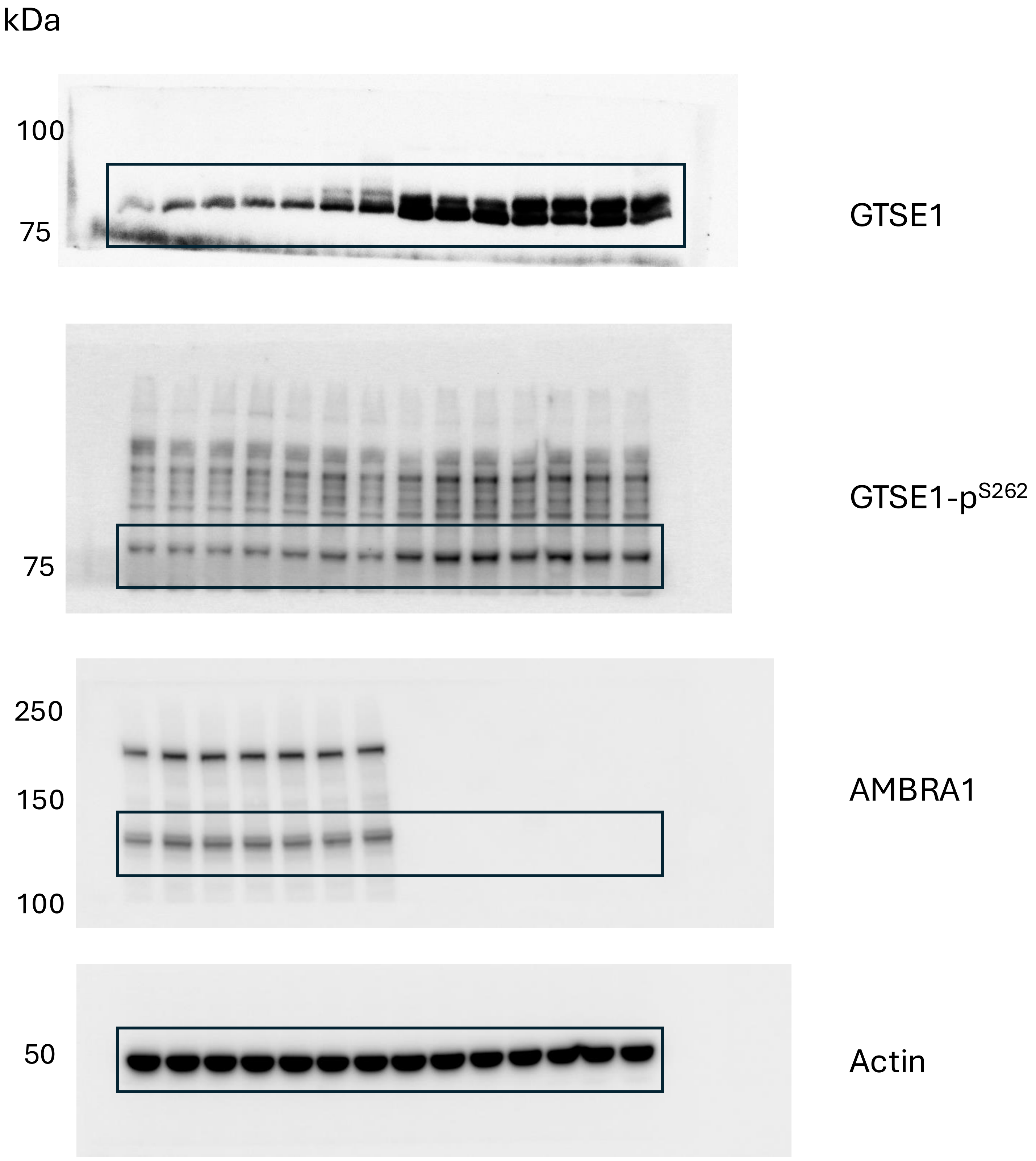

Figure 2H

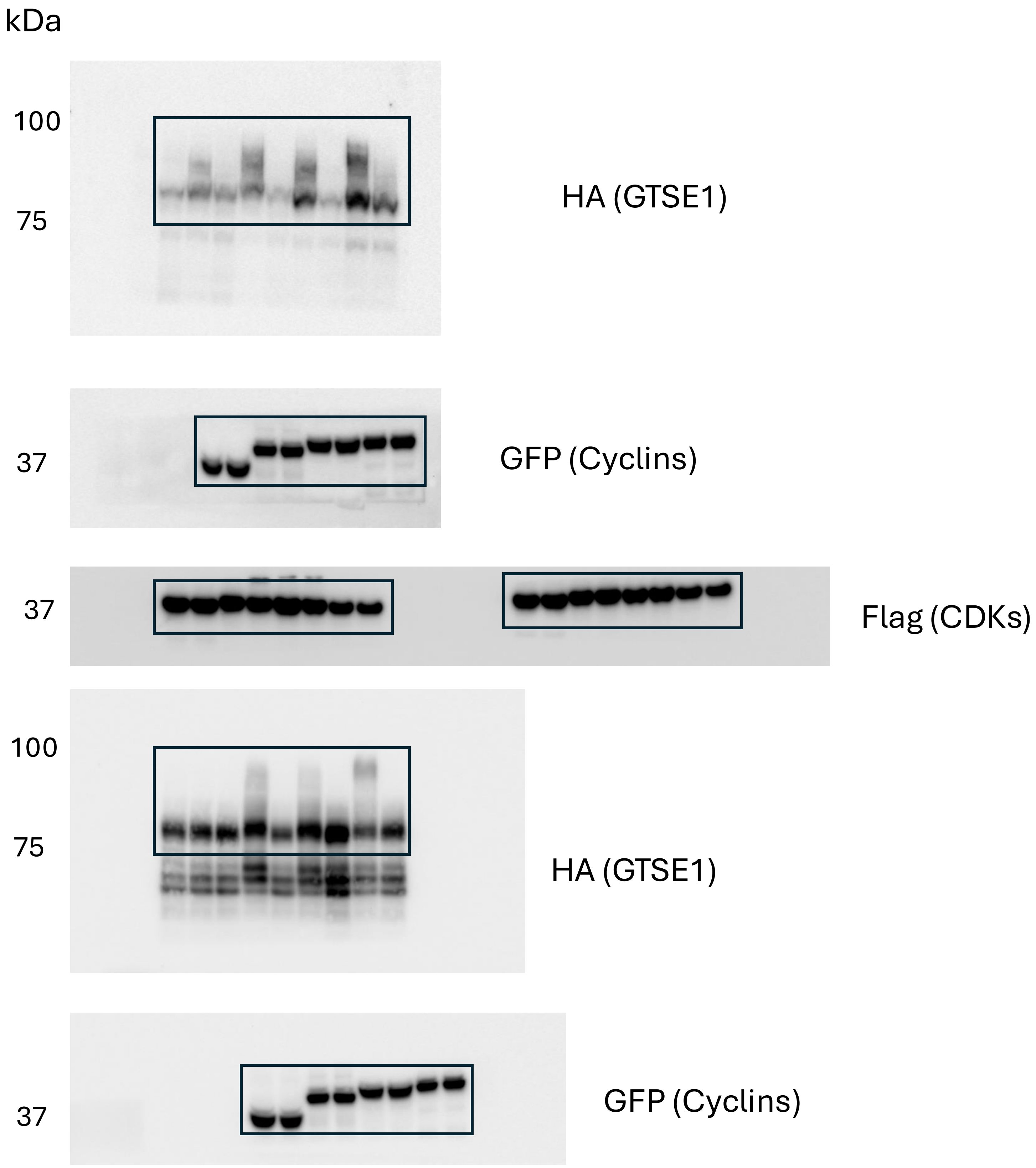
kDa
100
75
HA (GTSE1)
37
GFP (Cyclins)
37
Flag (CDKs)
100
75
HA (GTSE1)
37
GFP (Cyclins)
