# Supplementary material for: Stabilization of GTSE1 by cyclin D1–CDK4/6-mediated phosphorylation promotes cell proliferation with implications for cancer prognosis
Source: eLife. 2025 Apr 24;13:RP101075. doi: 10.7554/eLife.101075 (PMC12021411; doi:10.7554/eLife.101075)

Figure 3A

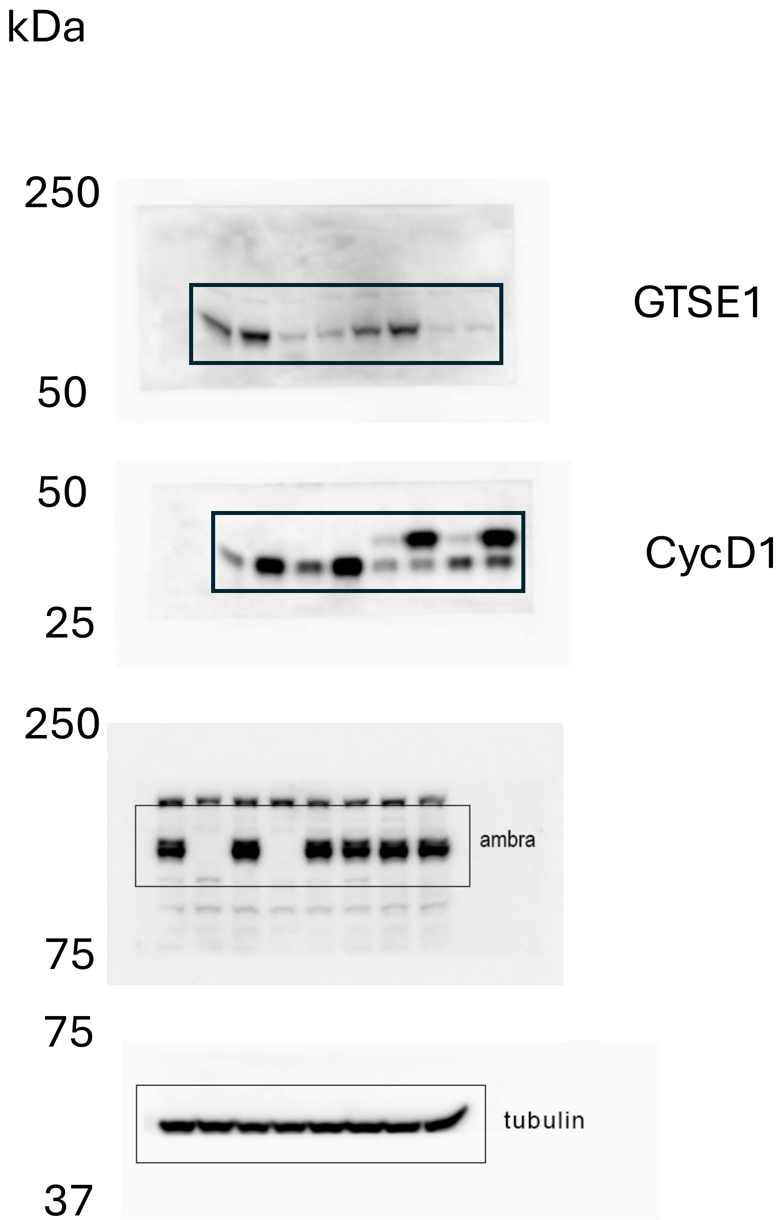

Figure 3B

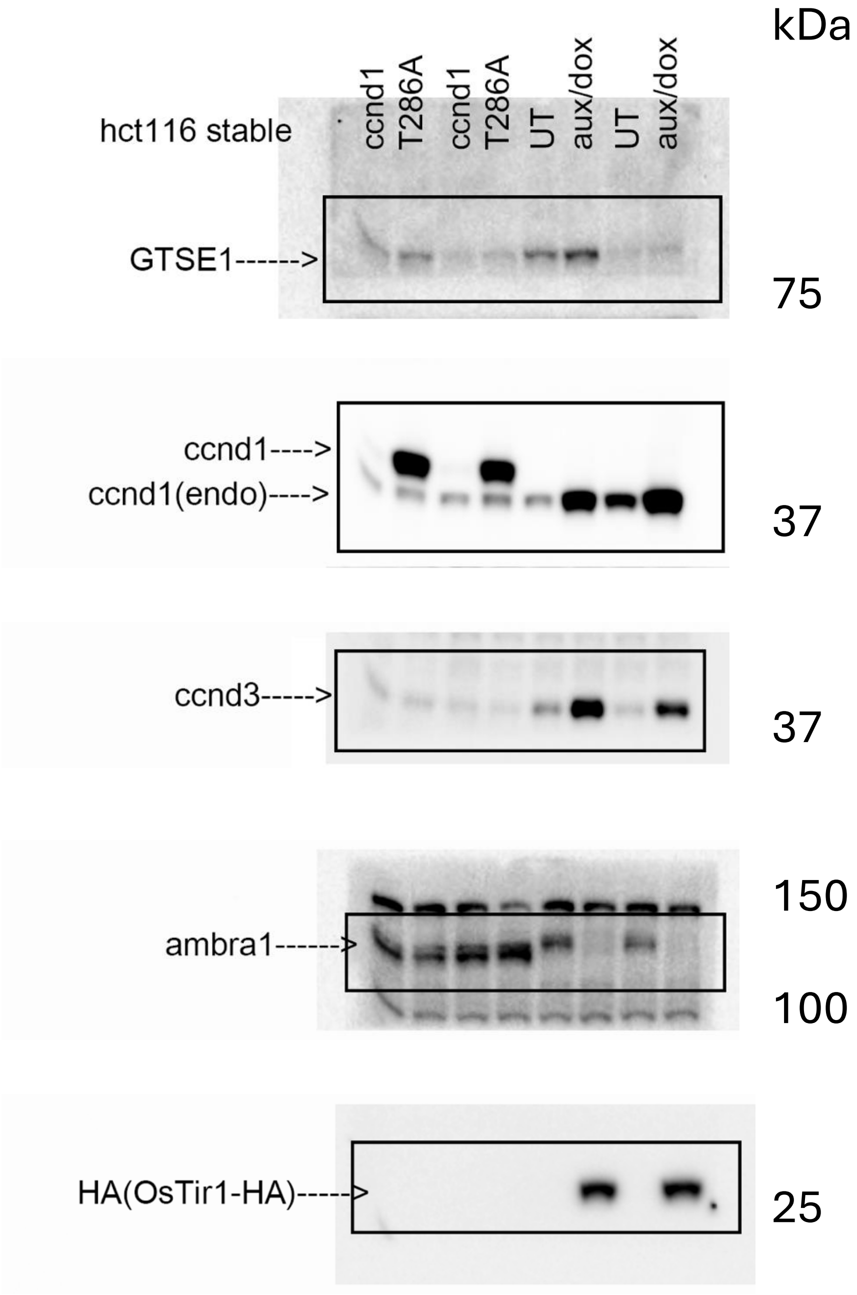
kDa
hct116 stable
ccnd1
T286A
ccnd1
T286A
UT
aux/dox
UT
aux/dox
GTSE1------->
75
ccnd1---->
ccnd1(endo)---->
37
ccnd3----->
37
150
ambra1------>
100
HA(OsTir1-HA)----->
25

Figure 3C

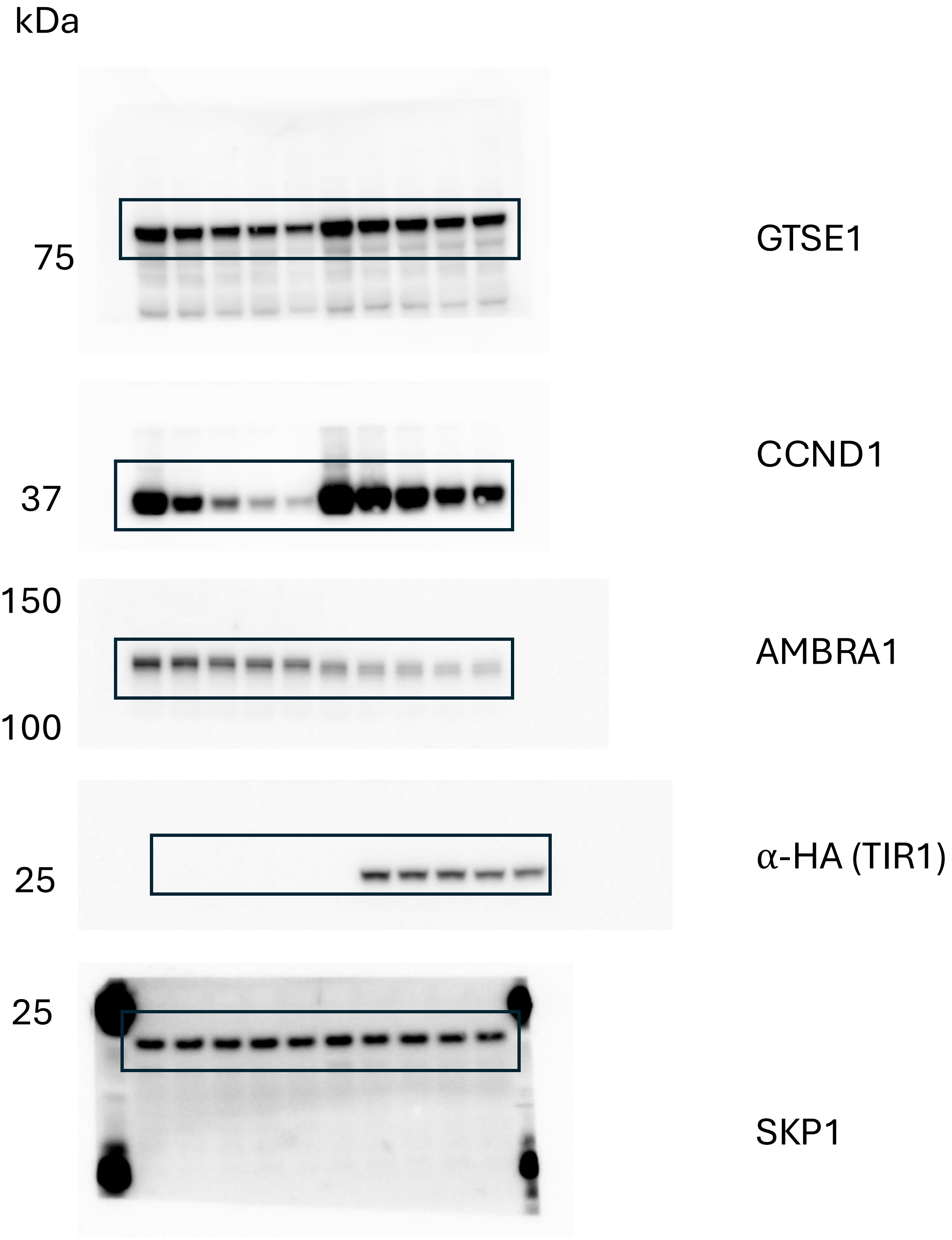

Figure 3D

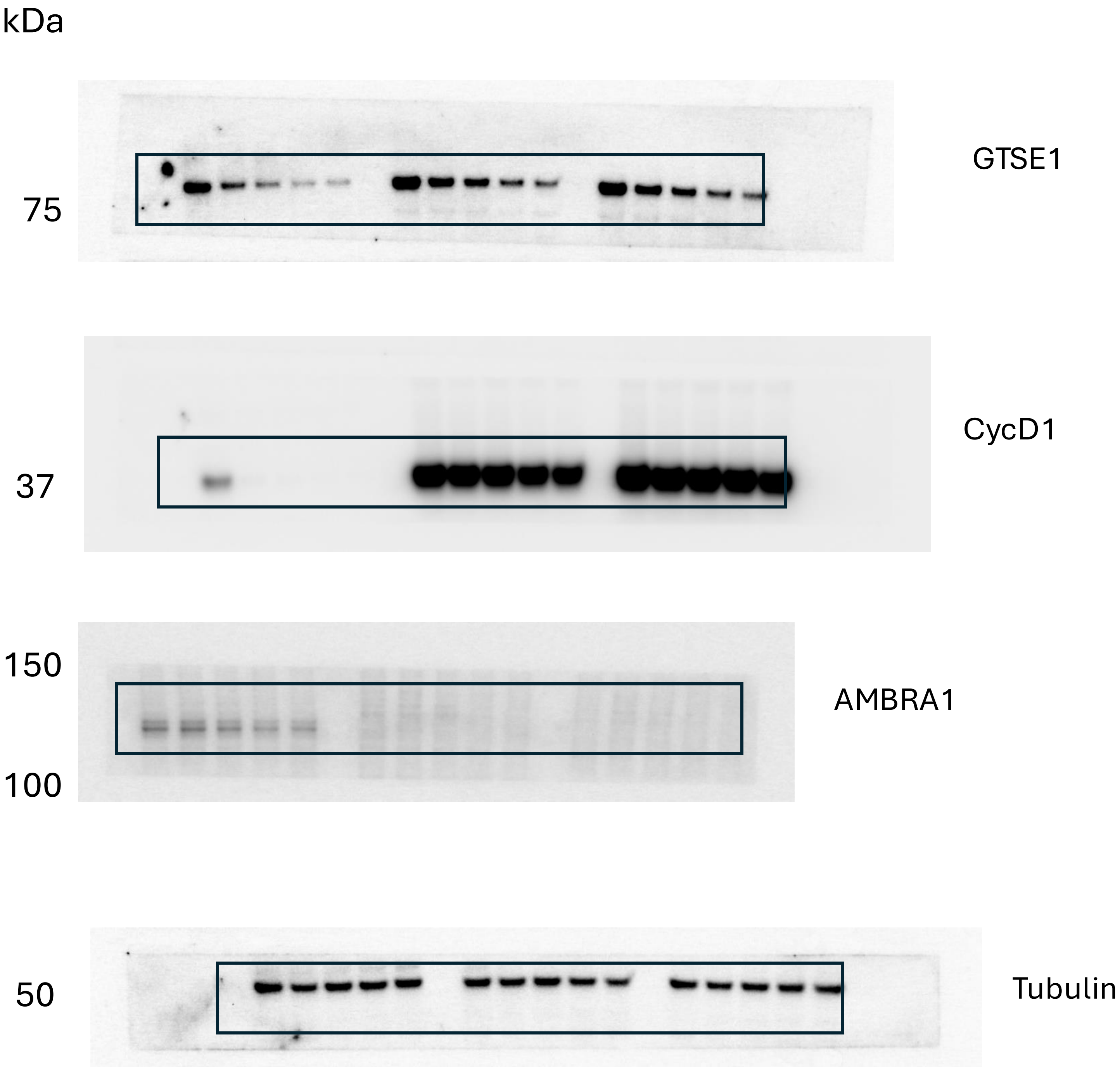

Figure 3E

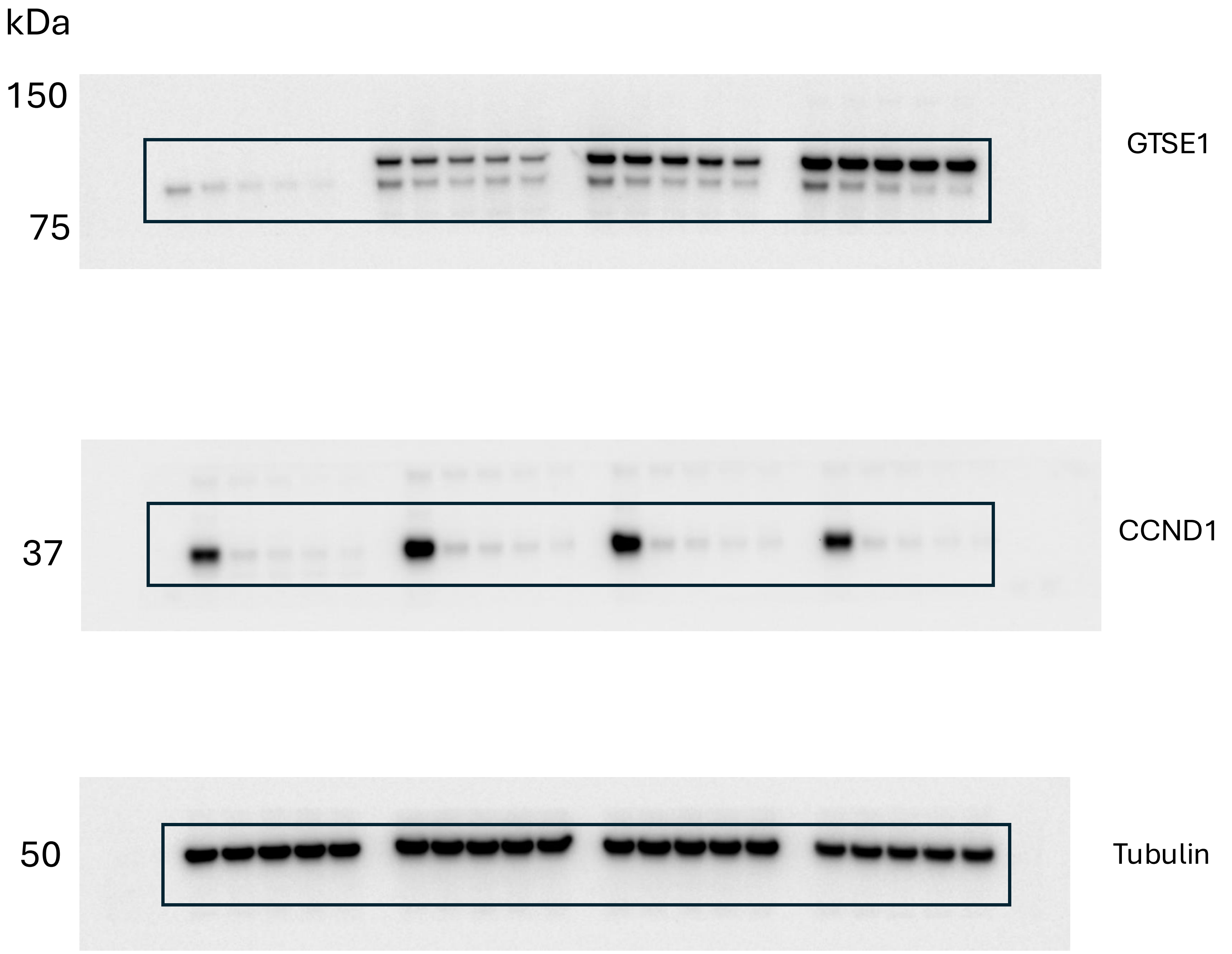

Figure 3I

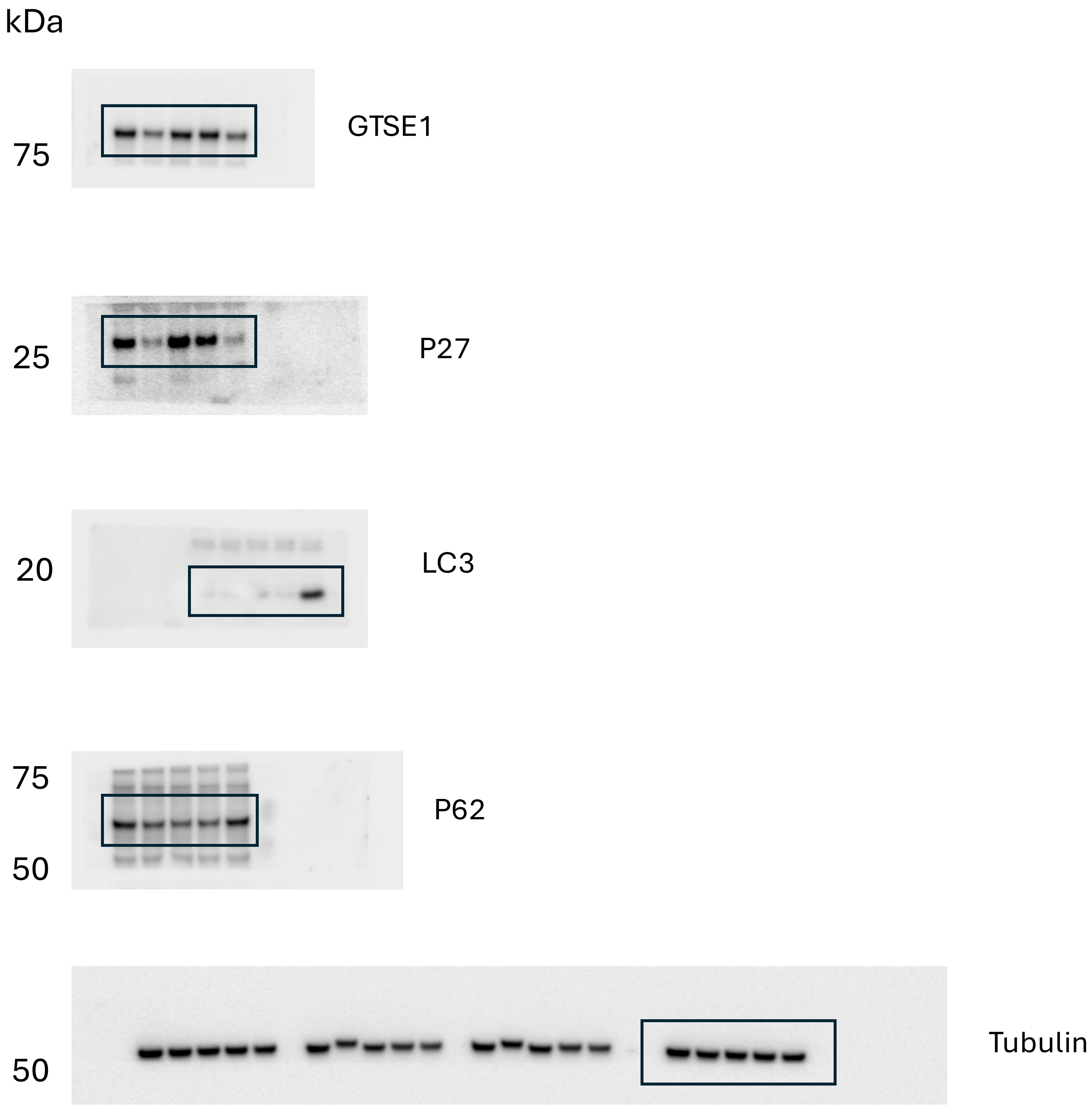

Supplement: Figure 3—source data 2. [file elife-101075-fig3-data2.pdf]
