# Supplementary material for: Stabilization of GTSE1 by cyclin D1–CDK4/6-mediated phosphorylation promotes cell proliferation with implications for cancer prognosis
Source: eLife. 2025 Apr 24;13:RP101075. doi: 10.7554/eLife.101075 (PMC12021411; doi:10.7554/eLife.101075)

Figure 3-figure supplement 1A

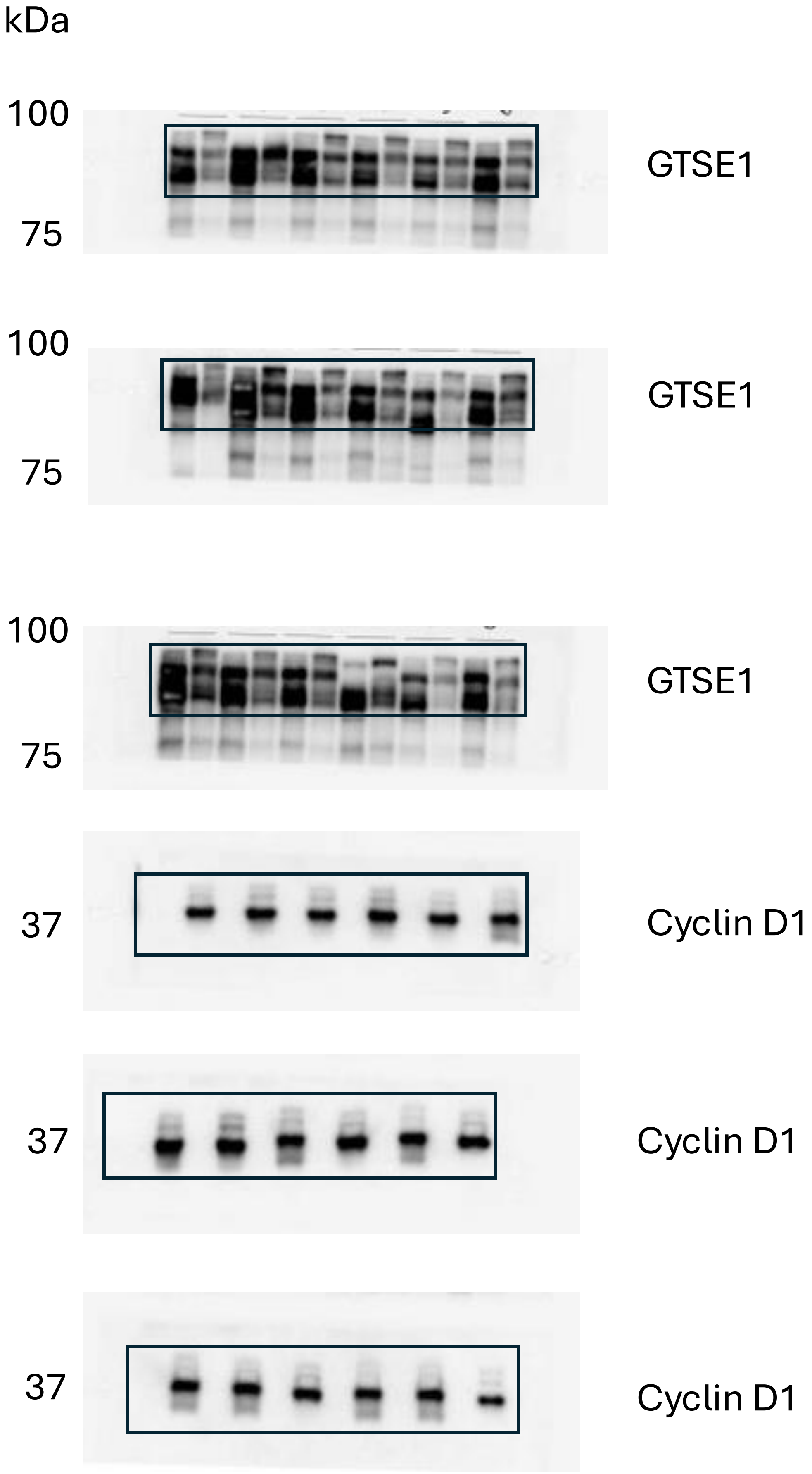

Figure 3-figure supplement 1B

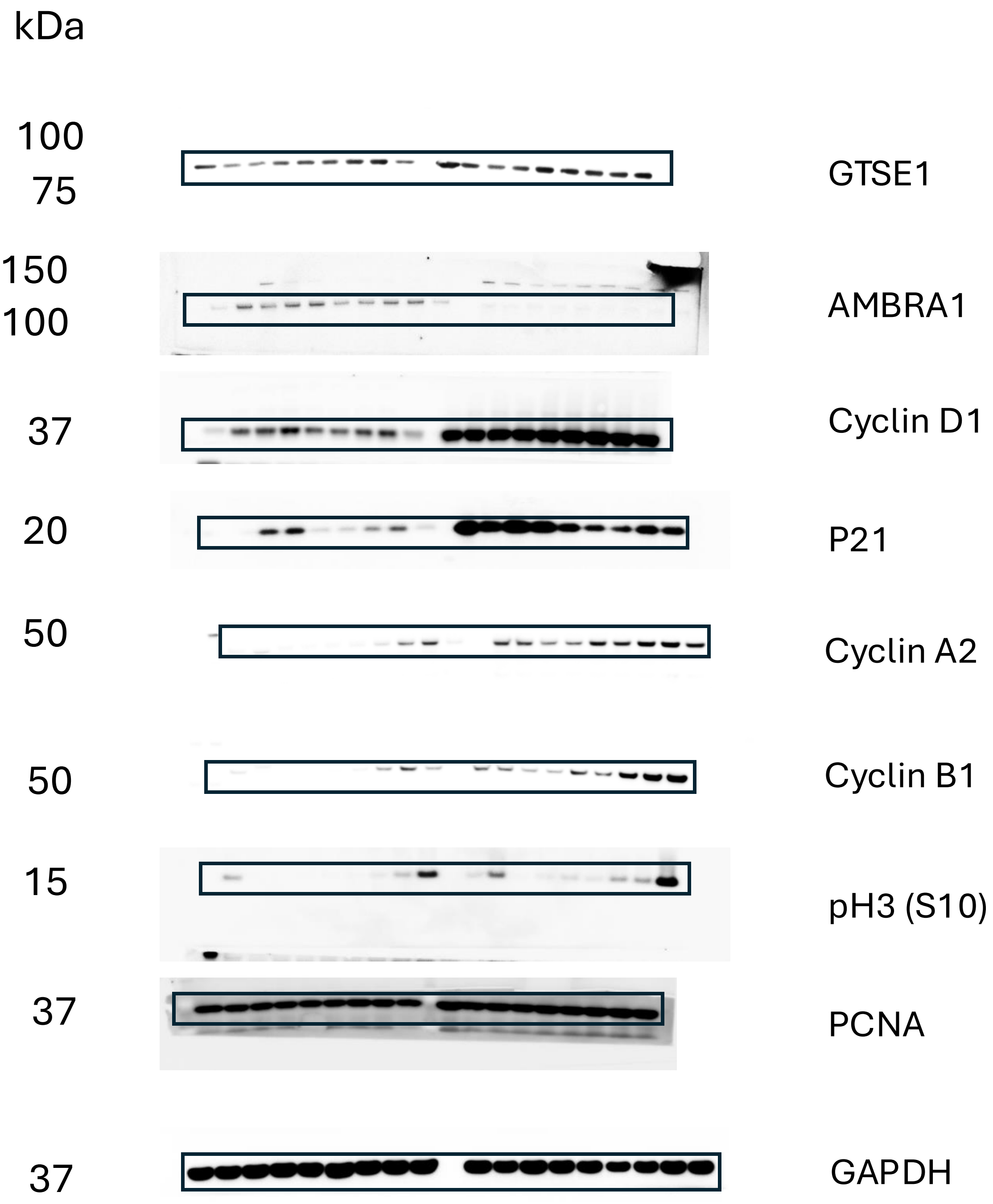

Figure 3-figure supplement 1C

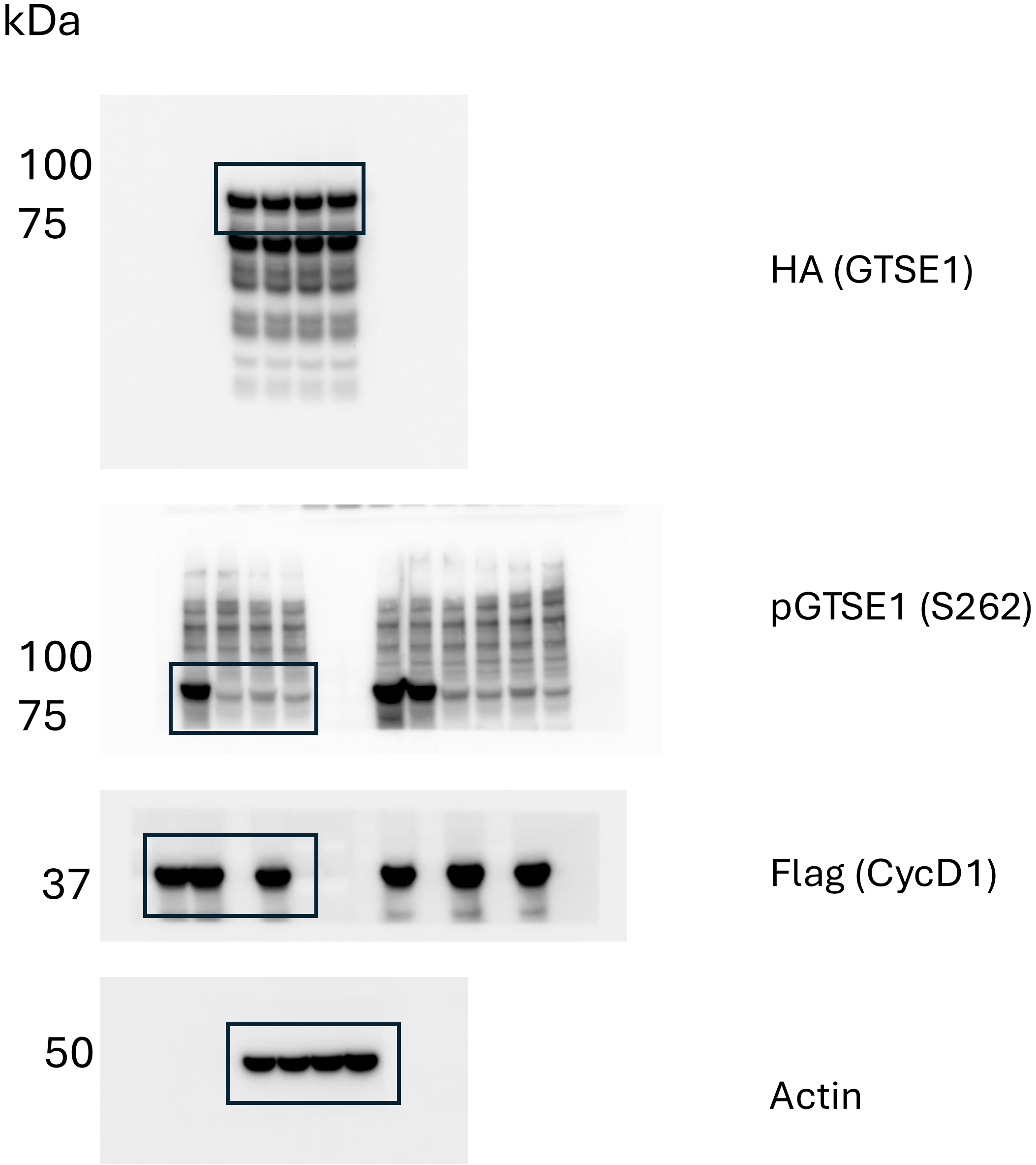

Figure 3-figure supplement 1E

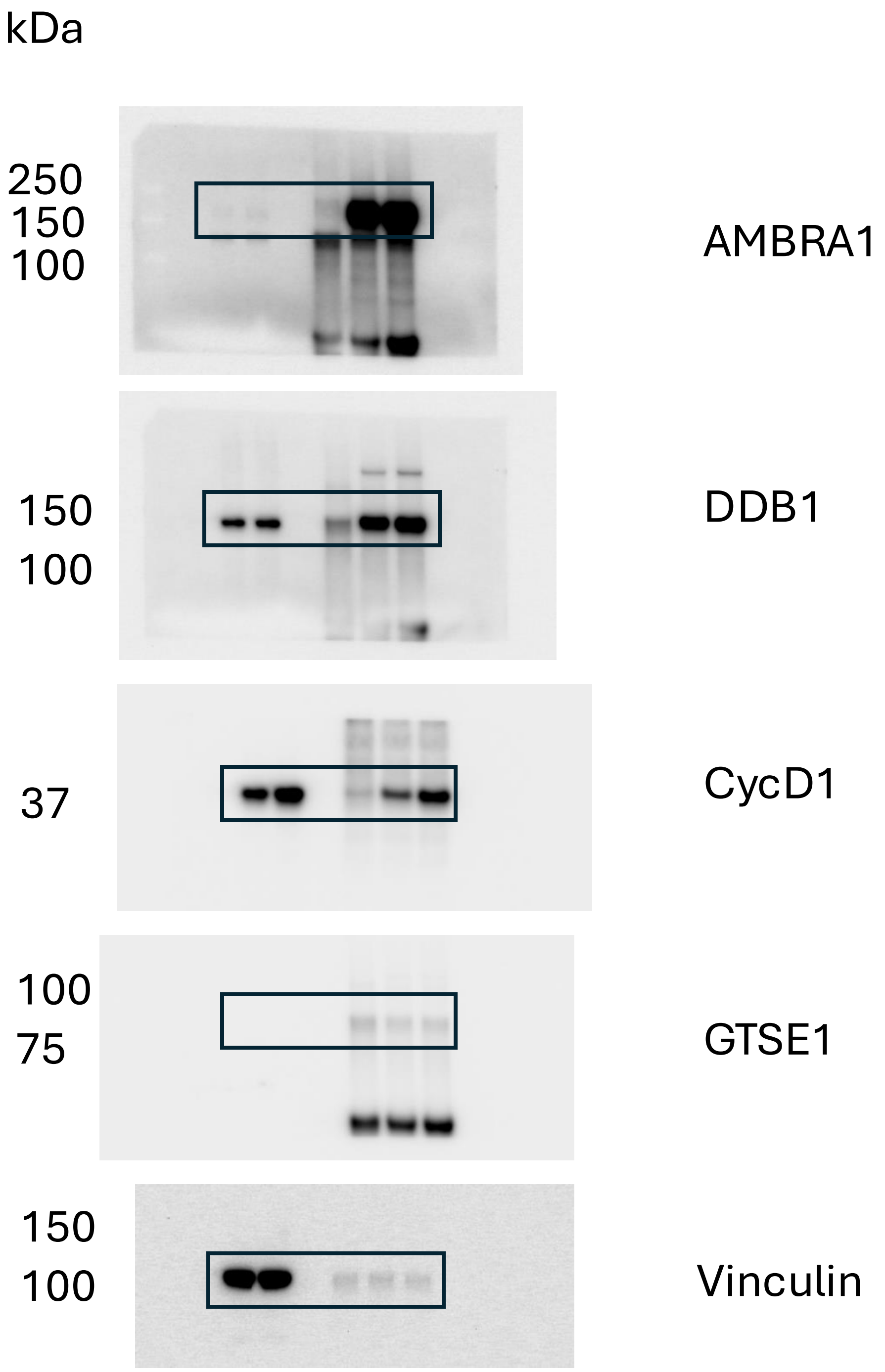

Supplement: Figure 3—figure supplement 1—source data 2. [file elife-101075-fig3-figsupp1-data2.pdf]
